# Six3 and Six6 jointly control diverse target genes in multiple cell populations over developmental trajectories of mouse embryonic retinal progenitor cells

**DOI:** 10.1371/journal.pone.0308839

**Published:** 2024-10-24

**Authors:** Alexander Ferrena, Xusheng Zhang, Rupendra Shrestha, Deyou Zheng, Wei Liu

**Affiliations:** 1 Department of Genetics, Albert Einstein College of Medicine, Bronx, New York, United States of America; 2 Department of Ophthalmology and Visual Sciences, Albert Einstein College of Medicine, Bronx, New York, United States of America; 3 The Ruth L. and David S. Gottesman Institute for Stem Cell and Regenerative Medicine Research, Albert Einstein College of Medicine, Bronx, New York, United States of America; Transilvania University of Brasov: Universitatea Transilvania din Brasov, ROMANIA

## Abstract

How tissue-specific progenitor cells generate adult tissues is a puzzle in organogenesis. Using single-cell RNA sequencing of control and Six3 and Six6 compound-mutant mouse embryonic eyecups, we demonstrated that these two closely related transcription factors jointly control diverse target genes in multiple cell populations over the developmental trajectories of mouse embryonic retinal progenitor cells. In the Uniform Manifold Approximation and Projection for Dimension Reduction (UMAP) graph of control retinas, naïve retinal progenitor cells had two major trajectories leading to ciliary margin cells and retinal neurons, respectively. The ciliary margin trajectory was from naïve retinal progenitor cells in the G1 phase directly to ciliary margin cells, whereas the neuronal trajectory went through an intermediate neurogenic state marked by *Atoh7* expression. Neurogenic retinal progenitor cells (Atoh7+) were still proliferative; early retinal neurons branched out from Atoh7+ retina progenitor cells in the G1 phase. Upon *Six3* and *Six6* dual deficiency, both naïve and neurogenic retinal progenitor cells were defective, ciliary margin differentiation was enhanced, and multi-lineage neuronal differentiation was disrupted. An ectopic neuronal trajectory lacking the Atoh7+ state led to ectopic neurons. Additionally, Wnt signaling was upregulated, whereas FGF signaling was downregulated. Notably, Six3 and Six6 proteins occupied the loci of diverse genes that were differentially expressed in distinct cell populations, and expression of these genes was significantly altered upon Six3 and Six6 dual deficiency. Our findings provide deeper insight into the molecular mechanisms underlying early retinal differentiation in mammals.

## Introduction

The formation, maintenance, and differentiation of tissue-specific progenitor cells are fundamental processes during organogenesis. Retinal development in vertebrates is an excellent model for dissecting these processes, and the mechanisms of retinal differentiation are harnessed for retinal regeneration to cure blindness. Specification of retinal progenitor cells starts with precursor cells in the eye field at the anterior neural plate [1]. Morphogenesis of the eye field leads to the evagination of optic vesicles, which later invaginate to form double-layered eyecups. This morphogenesis is coupled with cell fate specification. Upon the eyecup formation, which is on embryonic day 10.5 (E10.5) in mouse embryos, naïve progenitor cells for the neuroretinal epithelium and pigment epithelium are specified and compartmented into the inner and outer layers of the eyecup, respectively. Subsequently, the neuroretina is further patterned along the central-peripheral axis. In central regions close to the optic stalk, naïve retinal progenitor cells start neuronal differentiation by initiating the expression of basic helix loop helix (bHLH) transcription factor gene *Atoh7* as early as E11.5 in mice [2], leading to the generation of retinal ganglion cells as firstborn neurons in the retina. Retinal ganglion cell differentiation in the retina follows a central-to-peripheral wave under the regulation of FGF signaling in both chicks and mice [3, 4]. At the far periphery of the mouse retina, naïve neural retinal progenitor cells start to have restricted expression of ciliary margin markers *Otx1* and *Cdon* as early as E12.5 under the regulation of Wnt/β-catenin signaling [5, 6]. Opposing gradients of FGF and Wnt signaling regulate the central-peripheral patterning of the eyecup [7–9], but it is unclear how the signaling gradients are regulated.

Retinal progenitor cells possess remarkable multipotency as they generate all types of retinal neurons and Muller glia in the retina in an evolutionarily conserved temporal order [10]. Maintenance of a retinal progenitor cell pool and initiation of neuronal differentiation must be coordinated so that early- and late-born neurons are generated proportionally. Additionally, the competence of retinal progenitor cells changes over the course [11]. A few homeodomain and Sox family transcription factors are essential for multipotency since their deletions in mice (two paralogs in some cases) affect the competence of retinal progenitor cells. Pax6 is required for the multipotency of retinal progenitor cells through direct transcriptional activation of bHLH transcription factors [12]. Pax6 prevents the premature activation of a photoreceptor-differentiation pathway at the periphery whereas it maintains the multipotency of retinal progenitor cells in more central regions [13]. Sox2 is required for the retinal progenitor competence and ciliary margin-neuroretina boundary in a dose-dependent manner [14–16]. Meis1 and Meis2 are jointly required for the competence of retinal progenitor cells [17]. Despite these advances, the link between the formation and maintenance of multipotent retinal progenitor cells is largely unclear.

Our previous studies demonstrated that Six3 is required for neuroretinal specification because naïve retinal progenitor cells are absent and eyecups do not form when *Six3* is deleted at the anterior neural plate [18, 19]. After naïve retinal progenitor cells are specified, Six3 and Six6 are jointly required for the multipotency at the mid-periphery and the ciliary-margin suppression at the far periphery. The essential roles of Six3 and Six6 joint functions in the maintenance of multipotent retinal progenitor cells are consistent with the indispensable roles of Six3 in the specification of naïve retinal progenitor cells, providing a link between the specification and maintenance of retinal progenitor cells. Despite these advances, single-cell transcriptomes and cell trajectories regulated by Six3 and Six6 joint functions remain unclear; how naïve retinal progenitor cells are regulated to differentiate into ciliary and neuronal tissues along the central-peripheral axis in the retina is still an unsolved fundamental question in the field.

Single-cell RNA sequencing (scRNA-seq) is transformative in profiling cell states and trajectories in a heterogeneous cell population and is therefore instrumental in dissecting the mechanisms underlying organogenesis. scRNA-seq has been employed to study developing tissues, including the retina [20–25]. In scRNA-seq, individual cells are barcoded for transcriptomic profiling. Tens of thousands of cells are clustered using the k-Nearest Neighbor algorithm based on the distance between individual transcriptomes. Cluster markers are identified to define the expression signatures of cell types and states [26]. Differential expressed genes (DEGs) caused by gene inactivation are also identified. Additionally, cell trajectories are inferred using computational approaches, including RNA velocity analysis [27, 28].

In this study, we dissected the molecular mechanisms underlying the regulation of multipotent retinal progenitor cells using scRNA-seq profiling of Six3 and Six6 double knockout (*Six3*^*F/F*^ *Six6*^*−/−*^ *α-Cre*) and control eyecups. We revealed the expression signatures and developmental trajectories of naïve retinal progenitor cells in control retinas, discovered drastic defects in cell states and trajectories upon Six3 and Six6 dual deficiency, and identified diverse direct target genes of Six3 and Six6 in multiple cell populations, providing deeper insight into the molecular mechanisms underlying early retinal differentiation.

## Results

### Single-cell transcriptome profiling of Six3 and Six6 double knockout and littermate control eyecups for cell clustering

To elucidate the transcriptomes and cell trajectories jointly regulated by Six3 and Six6 in the maintenance and differentiation of multipotent retinal progenitor cells, we performed scRNA-seq of Six3 and Six6 double knockout (*Six3*^*F/F*^ *Six6*^*−/−*^ *α-Cre*, hereafter referred to as DKO) and littermate control (*Six3*^*+/−*^ *Six6*^*+/−*^) eyecups on embryonic day 13.5 (E13.5). Our previous studies indicated that the Cre activity, which was revealed by the R26R reporter, started in peripheral retinas at E10.5. In peripheral regions of DKO retinas, Six3 protein deletion was detected at E11.5, and alterations of gene expression started at E12.5 and became drastic at E13.5 [7]. Therefore, the stage of E13.5 is well suited for profiling the transcriptomes regulated by Six3 and Six6. Eyecups were used in this study since the removal of the lens inevitably damages ciliary margins. Approximately 10,000 cells from the DKO and control eyecups were captured using the Chromium fluid device for library preparation. Mapping of sequencing reads to mm10 using CellRanger revealed that control eyecups were sequenced for 10,315 cells at a depth of 21,726 reads / 2,619 genes per cell, and DKO eyecups were sequenced for 12,672 cells at a depth of 17,741 reads / 2,330 genes per cell.

**Fig 1:**
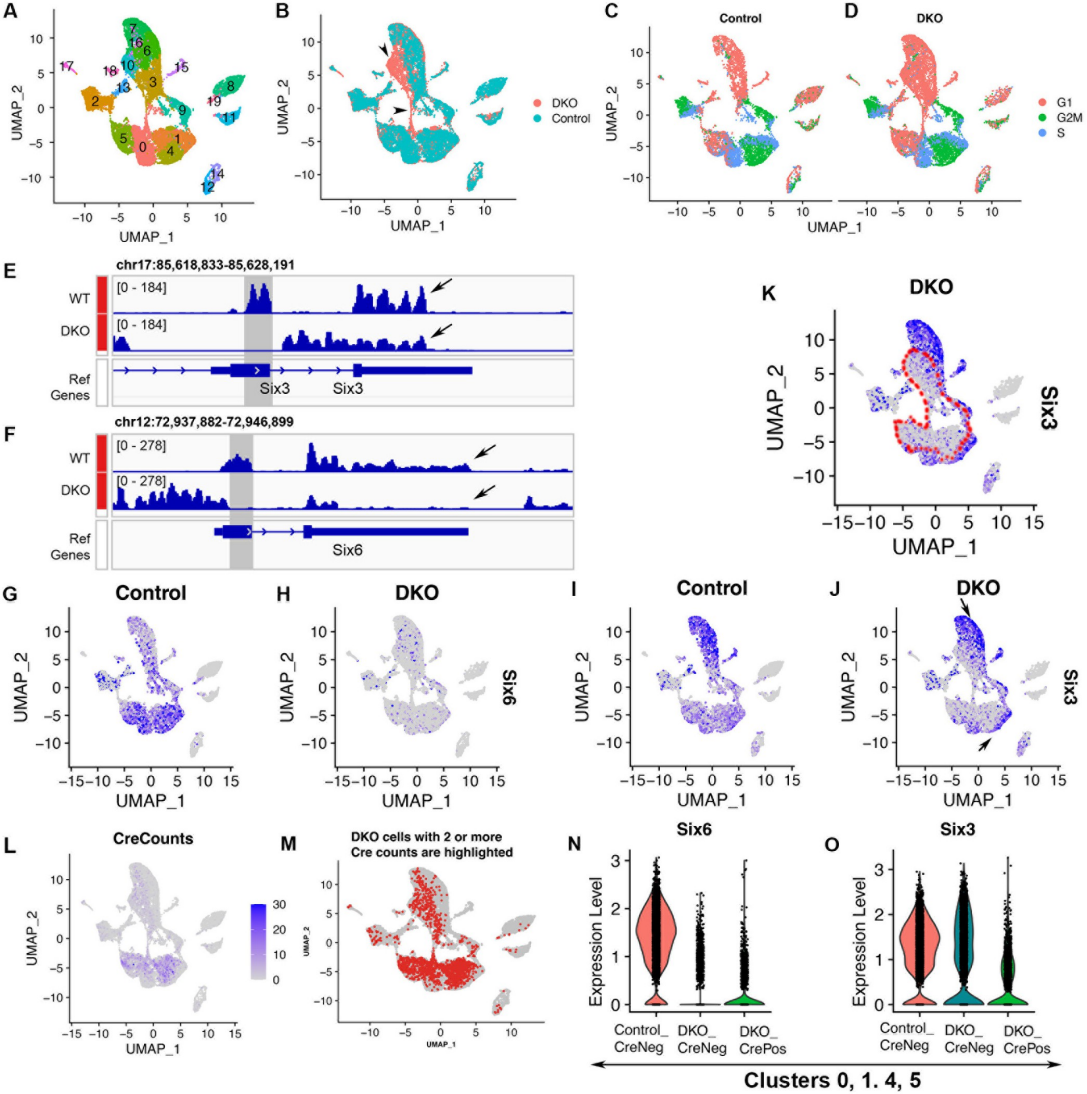
Cell clustering of the integrated scRNA-seq dataset and delineation of Six3- and Six6-deficient cells in the UMAP graph. Control and DKO eyecups at E13.5 were used for scRNA-seq; Seurat anchor-based integration was performed before cell clustering. **(A, B)** Clustering identified 20 cell clusters in the integrated dataset (A); cluster 10 only comprised cells from the DKO sample; a DKO-specific cell population comprising cluster 10 and subsets of clusters 0, 3, and 6 were identified (arrowheads in B). **(C, D)** Cell cycle phases were determined based on cell cycle scores. **(E, F)** Genome browser views of bulk RNA-seq of control and Six3 and Six6 compound mutant retinas demonstrated the deletions of targeted regions and the reductions of *Six6* and *Six3* mRNA levels (shown as the heights of read coverages) at 3’ regions (arrows in E, F). **(G, H)** *Six6* was widely expressed in retinal cells (G). Comparisons of *Six6* gene expression between control and DKO retinas indicated that *Six6* inactivation was reflected as reductions of mRNA expression levels in the feature plot (H). **(I–K)** *Six3* was widely expressed in retinal cells and largely overlapped with *Six6* expression. Comparisons of *Six3* gene expression between control and DKO retinas indicated that conditional *Six3* inactivation in DKO retinas was reflected as reductions of mRNA expression levels in well-delineated, inner areas in the UMAP graph (outlined areas in K). Six3-deficient (outlined areas in K) and Six3-wildtype-like cells (arrows in J, at the outer rim) were well separated in the UMAP graph of DKO retinas. **(L)** Counts of sequencing reads mapped to the Cre vector were high in clusters 0, 1, 4, and 5 and were moderate or low in other retinal clusters. **(M)** DKO cells with two or more Cre counts are highlighted (referred to as DKO_CrePos), and areas of these DKO_CrePos cells (M) matched the areas of DKO cells with strong reductions in *Six3* mRNA levels (K). **(N, O)** In clusters 0, 1, 4, and 5, reductions in *Six6* mRNA expression were indistinguishable between DKO_CrePos cells and DKO_CreNeg cells compared to control cells (N), consistent with germline *Six6* deletion; reductions in *Six3* mRNA expression were much stronger in DKO_CrePos cells than in DKO_CreNeg cells compared to control cells (O), consistent with conditional *Six3* deletion.

Quality control and data analysis were further performed using the Seurat package [26]. Cells were filtered (nFeature_RNA > 200 & nFeature_RNA < 6000 & percent.mt < 20), resulting in 9,822 and 12,146 cells for the control and DKO samples, respectively. The two datasets were normalized, their top variable features were found, and the Seurat anchor-based integration method was used to combine the datasets. Cell clustering of the integrated dataset identified 20 clusters and associated cluster markers (Fig 1; S1 and S2 Tables). DKO- and control-derived cells were found in all clusters except for cluster 10 (Fig 1A and 1B), which was exclusively composed of cells from DKO retinas. Notably, cluster 10, together with subsets of cells in clusters 0, 3, and 6, formed a cell population that only originated from the DKO sample, referred to as the DKO-specific cell population (arrowheads in Fig 1B), indicating ectopic cell identities caused by Six3 and Six6 dual deficiency. The ectopic cell population was in the G1 phase (Fig 1B and 1D), as determined using well-established cell cycle genes [29]. Identification of the DKO-specific cell population in the scRNA-seq dataset supports the drastic changes in gene expression caused by the Six3 and Six6 dual deficiency described in our previous study [7]. DKO retinas had a larger proportion of cells in the G1 phase and a smaller proportion of cells in the S phase compared with those in control retinas (S3 Table), indicating defects in cell cycle progression.

Non-retinal cells in the scRNA-seq dataset were identified based on cluster markers, which were identified when cells in one cluster were compared to cells in all the other clusters (S1 Fig, S2 Table). Clusters 8, 19, and 11 differentially expressed hemoglobin genes *Hba-X* and/or *Hba-a1*, indicating that these cells were red blood cells. Cluster 17 differentially expressed *C1qb* and *Tyrobp*, indicating that these cells were white blood cells. Clusters 12 and 14 differentially expressed *Cryab* and *Cryaa*, indicating that these cells were lens cells. These non-retinal clusters were well separated from other clusters in the UMAP graph and were not investigated any further.

Collectively, the scRNA-seq dataset provides an entry point for assessing the transcriptomes and developmental trajectories jointly regulated by Six3 and Six6 during early retinal differentiation.

### Identification of Six3- and Six6-deficient cells in the integrated scRNA-seq dataset

In DKO retinas, *Six3* was conditionally deleted in the peripheral regions by α-Cre whereas it remained in the central region; *Six6* was deleted in the germline [7]. Therefore, DKO retinas comprised both Six3-deficient and Six3-wildtype-like cells. We wondered whether cells with Six3 and Six6 dual deficiency in DKO retinas could be delineated in the scRNA-seq dataset so that transcriptomic changes could be correlated with Six3 and Six6 dual deficiency. In conditional *Six3* mutant mice, excision of the floxed DNA fragment in the *Six3* locus by the Cre recombinase leads to the deletion of exon 1, ablating its essential homeodomain [30]. In *Six6* mutant mice, the DNA fragment that encodes the essential homeodomain is replaced by a drug-selection cassette in the germline [31]. As expected, bulk RNA-seq of *Six3* and *Six6* compound-null retinas [7] demonstrated that the read coverage of targeted regions in *Six3* and *Six6* loci was absent. Importantly, the read coverage of transcripts, including the 3’ regions of mRNA that were measured by scRNA-seq, was substantially reduced (Fig 1E and 1F). In the feature plots of the scRNA-seq dataset, *Six6* mRNA was widely expressed in control retinas, but it was strongly reduced in DKO retinas (Fig 1G and 1H). *Six3* was widely expressed in retinal cells and largely overlapped with *Six6* (Fig 1G and 1I). When *Six3* expression in the control and DKO retinas was compared in the UMAP graphs, areas with strong reductions in *Six3* mRNA levels were found in the inner regions of the UMAP graph of DKO retinas (Fig 1I–1K, outlined in Fig 1K), indicating that cells in these areas were deficient for Six3. In contrast, *Six3* expression remained at the outer edge of the UMAP graph of DKO retinas (arrows in Fig 1J), indicating that these cells were Cre-negative cells in DKO retinas.

We identified Cre-positive and Cre-negative retinal cells in the DKO sample by measuring the expression of the Cre vector. Counts of sequencing reads mapped to the Cre vector were high in clusters 0, 1, 4, and 5 and were moderate or low in clusters 9, 3, 10, 6, 16, and 7 (Fig 1L). In quality-filtered DKO cells, there were 2038 retinal cells with two or more Cre counts (referred to as DKO_CrePos hereafter) and 8944 retinal cells with less than two or no Cre counts (referred to as DKO_CreNeg cells hereafter). Notably, the areas of DKO_CrePos cells as a whole matched the areas of those cells with strong reductions in *Six3* mRNA expression (Fig 1I, 1J and 1M), indicating that the Cre activity was highly concordant with conditional *Six3*-deletion. The areas of DKO_CrePos cells comprised most areas of DKO retinal cells in the UMAP graph, indicating that the percentage of conditional *Six3* deletion was high. Cre expression in some DKO cells was dropped out, a common issue in scRNA-seq. Nevertheless, DKO_CrePos cells were proved to be sufficient for differential gene expression analysis (see also S4–S7 Tables).

We examined *Six6* and *Six3* mRNA expression using violin plots. In clusters 0, 1, 4, and 5, reductions in *Six6* expression were indistinguishable between DKO_CreNeg and DKO_CrePos cells compared with control cells (Fig 1N), consistent with *Six6* germline deletion; reductions in *Six3* expression were much stronger in DKO_CrePos cells than in DKO_CreNeg cells compared with control cells (Fig 1O), consistent with conditional *Six3* deletion.

Collectively, the inactivation of *Six3* and *Six6* was reflected by reductions in their mRNA levels in both bulk and scRNA-seq. Six3-deficient and Six3-wildtype-like cells were well separated in the UMAP graph, leading to the delineation of Six3-deficient cells in DKO retinas. Those cells from DKO retinas that showed significant reductions of *Six3* mRNA expression compared with control retinas in the UMAP graphs were deficient for both Six3 and Six6 and were the focus of this study. For clarity, Six3- and Six6-deficient cells or dual deficiency are referred to those cells showing reductions in both *Six3* and *Six6* mRNA levels in feature plots, representing simultaneous *Six3* conditional inactivation by α-Cre and *Six6* germline inactivation; DKO cells are referred to cells that are originated from the DKO sample. The DKO-specific cell population was a subset of Six3- and Six6-deficient cells (Fig 1B and 1K), indicating that the DKO-specific cell population was caused by Six3 and Six6 dual deficiency.

### Retinal progenitor cells comprise naïve and neurogenic populations in control retinas and both cell populations are defective upon Six3 and Six6 dual deficiency

**Fig 2:**
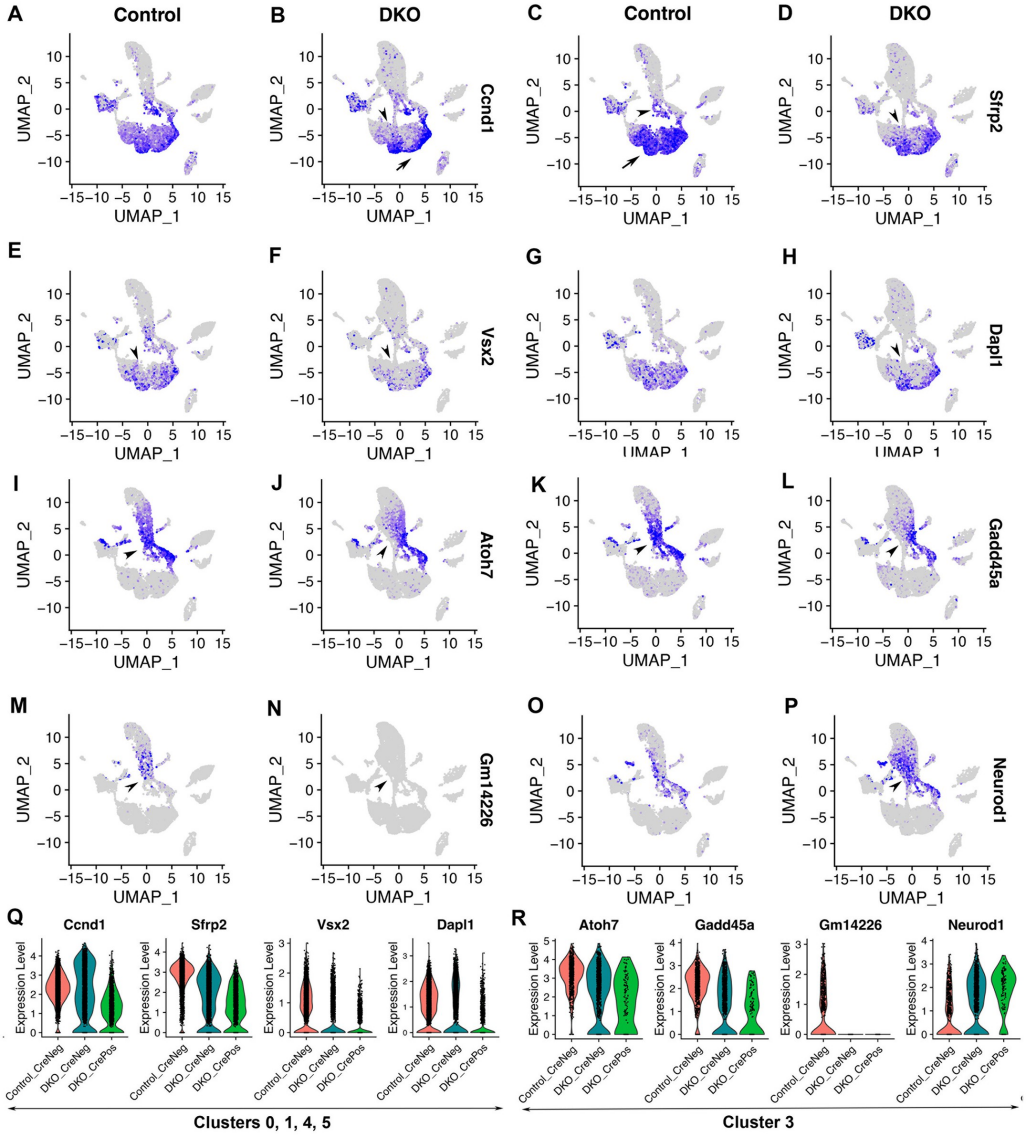
Retinal progenitor cells comprise naïve and neurogenic populations in control retinas and both cell populations are defective upon Six3 and Six6 dual deficiency. See Fig 1 for the information on cell clusters, cell cycle phases, and Six3 and Six6 dual deficiency. **(A–H)** In control retinas, clusters 0, 1, 4, and 5 differentially expressed *Ccnd1*, *Sfrp2*, and *Vsx2* (A, C, E, respectively; see also S2 Table), indicating that these cells were naïve retinal progenitor cells. These naïve retinal progenitor cells also differentially expressed *Dapl1* (G). Cluster 2 also expressed these naïve retinal progenitor cell markers. However, cluster 2 was marked by negative gene markers (see also S2 Fig; see also S2 Table) and had lower values for nCount_RNA and nFeature_RNA (see also S3 Fig), indicating differences between cluster 2 and clusters 0, 1, 4, and 5. In Six3- and Six6-deficient cells, the expression of naïve retinal progenitor cell markers was significantly reduced (arrowheads in B, D, F, and H; see also S4 Table). **(K–T)** In control retinas, clusters 9 and 3 differentially expressed *Atoh7* (I; see also S2 Table), indicating that these cells were neurogenic retinal progenitor cells. Naïve and neurogenic retinal progenitor cells were well segregated based on *Atoh7* expression (I). Neurogenic retinal progenitor cells also differentially expressed marker genes *Gadd45a* (K) and *Gm14226* (M). Subsets of neurogenic retinal progenitor cells also expressed *Neurod1* (I, O). In Six3- and Six6-deficient cells, the expression of *Atoh7*, *Gadd45a*, and *Gm14226* was significantly downregulated (arrowheads in J, L, N), whereas the expression of *Neurod1* was significantly upregulated (P; see also S5 Table). **(Q, R)** Expression changes in these genes were also shown in violin plots.

We then assessed the identities of retinal cell clusters. Cluster markers were identified by comparing one cluster with all other clusters (S2 Table). In control retinas, clusters 0, 1, 4, and 5 differentially expressed *Ccnd1*, *Sfrp2*, and *Vsx2* (Fig 2A, 2C and 2E; S2 Table), which are well-established gene markers of naïve retinal progenitor cells. These clusters also differentially expressed *Dapl1* based on our scRNA-seq data (Fig 2G; S2 Table). Clusters 0, 1, 4, and 5 were grossly aligned with the cell cycle phases (Fig 1A and 1C), indicating that naïve retinal progenitor cells were further clustered based on cell cycle stages. In contrast to naïve retinal progenitor cells, clusters 9 and 3 highly expressed *Atoh7* (Fig 2I; S2 Table), indicating their neurogenic identities. Clusters 9 and 3 were aligned with cell cycle phases, and Atoh7+ neurogenic retinal progenitor cells were still proliferative (Figs 1A and 1C; 2I). A small portion of Atoh7+ retinal progenitor cells also expressed *Ccnd1*, *Sfrp2*, and *Vsx2* (Fig 2A, 2C, 2E and 2I; the positions were indicated by arrowheads in Fig 2C and 2I), indicating that these cells were transitioning from the naïve retinal progenitor state to the neurogenic progenitor state. *Gadd45a* and *Gm14226* largely overlapped with *Atoh7* in neurogenic retinal progenitor cells (Fig 2I, 2K and 2M; S2 Table). A subset of Atoh7+ neurogenic retinal progenitor cells also expressed *Neurod1* (Fig 2I and 2O; S2 Table).

We then identified DEGs caused by Six3 and Six6 dual deficiency in naïve and neurogenic retinal progenitor cells by comparing DKO_CrePos cells with control cells in clusters 0, 1, 4, and 5 (S4 Table) and in clusters 3 and 9 (S5 Table). Upon Six3 and Six6 dual deficiency, significant reductions in gene expression were found for *Ccnd1* (Fig 2B; avg_log2FC = -1.3, p_val_adj = 0, S4 Table), *Sfrp2* (Fig 2D; avg_log2FC = -1.9, p_val_adj = 0, S4 Table), *Vsx2* (Fig 2F; avg_log2FC = -1.0, p_val_adj = 1.69E-209, S4 Table), *Dapl1* (Fig 2H; avg_log2FC = -1.1, p_val_adj = 6.05E-238, S4 Table), *Atoh7* (Fig 2J; avg_log2FC = -1.3, p_val_adj = 8.51E-34, S5 Table), *Gadd45a* (Fig 2L; avg_log2FC = -1.4, p_val_adj = 4.06E-42, S5 Table), *Gm14226* (Fig 2N; avg_log2FC = -1.2, p_val_adj = 2.36E-15, S5 Table), and whereas a significant increase in expression was found for *Neurod1* (Fig 2P; avg_log2FC = 0.85, p_val_adj = 4.98E-16, S5 Table). Expression changes in these genes were also demonstrated in violin plots (Fig 2Q and 2R). Neurod1 is a pan-amacrine transcription factor in the mouse retinas [32], and its upregulation in the scRNA-seq dataset was consistent with the increased number of amacrine cells in DKO retinas described in our previous study [7].

Taken together, naïve and neurogenic retinal progenitor cells form two distinct cell populations in the UMAP graph in the control; both cell populations are defective upon Six3 and Six6 dual deficiency.

We noted that cluster 2 also expressed naïve retinal progenitor cell markers (Figs 1A, 2A–2H) and cluster 13 also expressed neurogenic markers (Figs 1A; 2I–2P) in both control and DKO retinal cells, but these two clusters were separated from the main groups of retinal progenitor cells. Compared with clusters 5, 0, 4, 1, 9, and 3, clusters 2 and 13 were defined by negative marker genes, including *Kcnq1ot1*, *mt-Co1*, *mt-Nd2*, and *mt-Nd4* (S2 Fig; S2 Table). Interestingly, clusters 2 and 13 had lower values of nCount_RNA, nFeature_RNA, and percent.mt (S3 Fig). Therefore, clusters 2 and 13 were separate retinal cell populations that had lower counts of RNA molecules and genes, perhaps due to lower rates of RNA capture for unknown technical reasons. Alternatively, the low counts of RNA molecules and genes in clusters 2 and 13 may reflect uncharacterized retinal cells that have a lower number of expressed genes. In support of this notion, nFeature_RNA varied significantly along cell clusters; clusters 8 and 11, which were red blood cells, also had low counts of nFeature_RNA as expected. Further studies are needed to determine why clusters 2 and 13 were separated from the main groups of retinal cells in the UMAP graph. Since clusters 2 and 13 were not within Six3 and Six6 deficient cells, these two clusters were not the focus of our study.

### Early retinal neurons branch out from Atoh7+ neurogenic retinal progenitors in the UMAP of control retinas and these early retinal neurons are defective upon Six3 and Six6 dual deficiency

**Fig 3:**
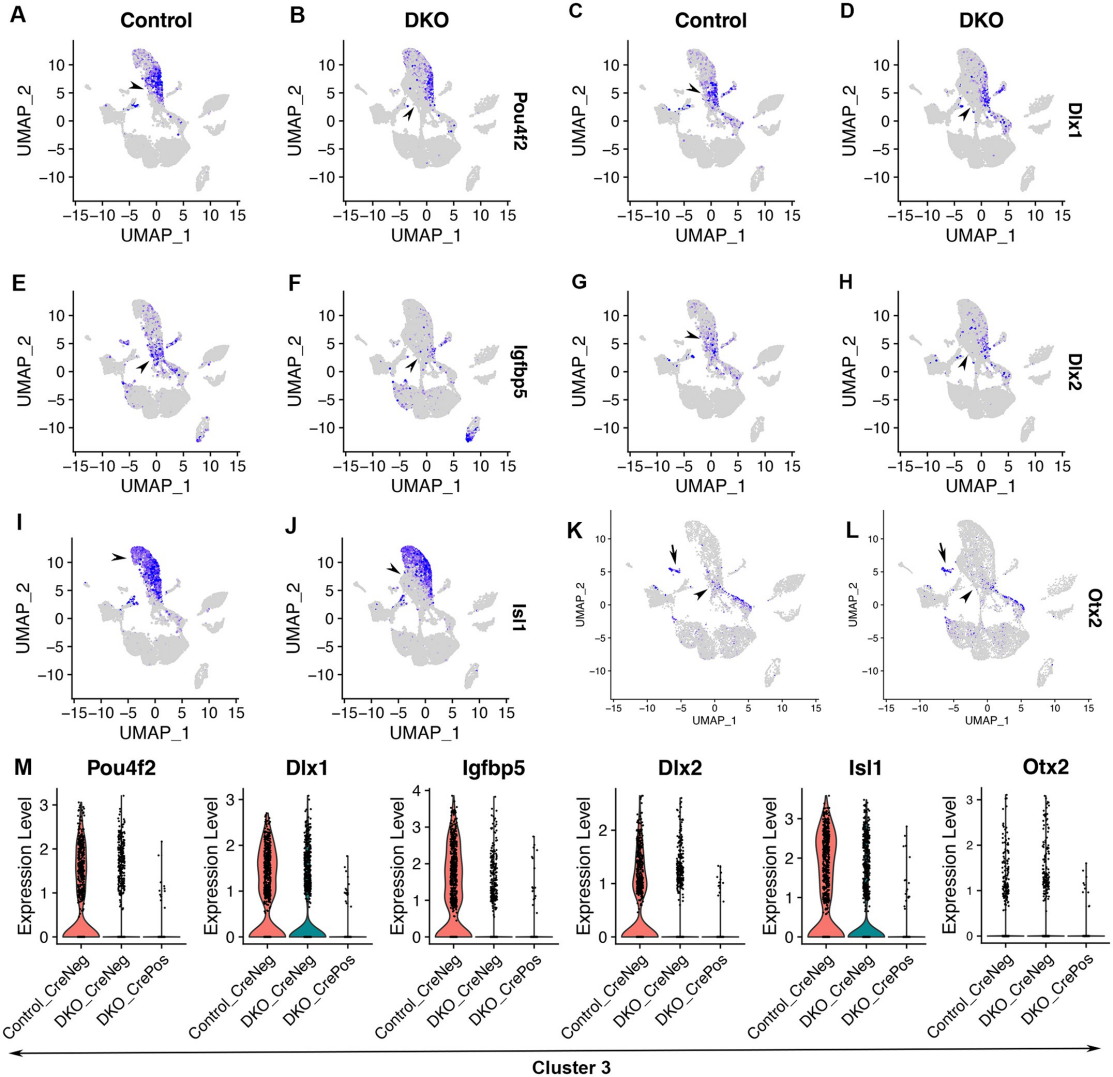
Early retinal neurons branch out from Atoh7+ neurogenic retinal progenitors in the UMAP of control retinas and these early retinal neurons are defective upon Six3 and Six6 dual deficiency. See Fig 1 for the information on cell clusters, cell cycle phases, and Six3 and Six6 dual deficiency. **(A–J)** In control retinas, early retinal ganglion cells were marked by the expression of *Pou4f2*, *Dlx1*, *Igfbp5*, *Dlx2*, and *Isl1* (arrowheads in A, C, E, G, I; see also S2 Table). Expression patterns of *Atoh7*, *Pou4f2*, and *Isl1* in the UMAP graph indicated a progression of gene expression starting from neurogenic retinal progenitor cells toward retinal ganglion cells at advanced stages. In Six3- and Six6-deficient cells, *Pou4f2*, *Dlx1*, *Igfbp5*, *Dlx2*, and *Isl1* expression was significantly downregulated (B, D, F, H, J; see also S5 Table). **(K, L)** In control retinas, *Otx2* was expressed in early photoreceptor cells (cluster 18; arrow in K) and a subset of neurogenic retinal progenitor cells (arrowhead in K). Upon Six3 and Six6 dual deficiency, *Otx2* expression was significantly downregulated in neurogenic retinal progenitor cells (arrowheads in K, L; *Otx2* violin plot was in Fig 3M; see also S5 Table). **(M)** Expression changes in these genes were also shown in violin plots.

We assessed the differentiation of early retinal neurons in the scRNA-seq dataset. In the control, clusters 3 highly expressed retinal ganglion cell markers *Pou4f2*, *Dlx1*, *Igfbp5*, and *Dlx2*, and clusters 3, 6, 7, and 16 highly expressed *Isl1* (Fig 3A, 3C, 3E, 3G and 3I; S2 Table). The expression of these genes overlapped with *Atoh7* expression in cluster 3 (compare Fig 3A, 3C, 3E and 3G, and 3I with Fig 2I). Additionally, *Isl1* expression started in cluster 3, through cluster 6, and continued to cluster 7, which was the end of this branch (Fig 3I). The expression patterns of *Sfrp2*, *Atoh7*, *Pou4f2*, and *Isl1* in the UMAP graph revealed progressively advancing states toward retinal ganglion cell differentiation in control retinas (Figs 2C, 2I, 3A and 3I). Therefore, the UMAP captures a progression of cell states starting from naïve retinal progenitors, through neurogenic progenitors, and to retinal ganglion cells at advanced stages.

We identified DEGs caused by Six3 and Six6 dual deficiency during retinal ganglion cell differentiation by comparing DKO_CrePos cells with control cells in clusters 10, 6, and 3 (S6 Table). In Six3- and Six6-deficient cells, significant expression changes were found for *Pou4f2* (Fig 3B; avg_log2FC = -1.6, p_val_adj. = 2.51E-44, S6 Table), *Dlx1* (Fig 3D; avg_log2FC = -1.3, p_val_adj. = 1.34E-39, S6 Table), *Igfbp5* (Fig 2F; avg_log2FC = -1.6, p_val_adj. = 1.99E-30, S6 Table), *Dlx2* (Fig 3H; avg_log2FC = -0.87, p_val_adj. = 1.26E-20, S6 Table), and *Isl1* (Fig 3I; avg_log2FC = -1.8, p_val_adj. = 5.79E-50, S6 Table). Expression changes in these genes were also demonstrated in violin plots (Fig 3M). These findings indicate that early retinal ganglion cell differentiation is defective upon Six3 and Six6 dual deficiency.

The differentiation of photoreceptor cells was also defective upon Six3 and Six6 dual deficiency. In control retinal cells, cluster 18 was marked by *Otx2* expression (Figs 1A and 3K). Interestingly, *Otx2* was also expressed in a subset of neurogenic retinal progenitor cells in the control (arrowhead in Fig 3K). Upon Six3 and Six6 dual deficiency, *Otx2* mRNA expression was downregulated (arrowheads in Fig 3K and 3L; Otx2 panel in 3M; avg_log2FC = -0.68, p_adj_FDR = 2.21E-05, S5 Table), indicating defects in photoreceptor cell differentiation.

Collectively, early retinal neurons, which are dominated by retinal ganglion cells at E13.5, branch out from Atoh7+ neurogenic retinal progenitor cells in the G1 phase in the UMAP graph of control retinas; differentiation of these early retinal neurons is defective upon Six3 and Six6 dual deficiency.

### The DKO-specific cell population is ectopic and featured by gene markers for the forebrain and amacrine cells

The DKO-specific cell population was ectopic since there were no control cells in this area of the UMAP graph (arrowheads in Fig 1A and 1B). The DKO-specific cell population comprised cluster 10 and subsets of cells in clusters 0, 3, and 6; they barely expressed neurogenic progenitor marker genes *Atoh7*, *Gadd45a*, and *Gm14226* (Fig 2I–2N) and retinal ganglion cell markers *Pou4f2*, *Dlx1*, *Igfbp5*, and *Dlx2* (Fig 3A–3J). Cluster 10, a DKO-specific cluster, was close to cluster 6 in the UMAP graph. Additionally, both the PCA analysis of cluster average gene expression and hierarchical clustering of the expression correlation matrix showed that cluster 10 was close to cluster 6 (S4 Fig).

**Fig 4:**
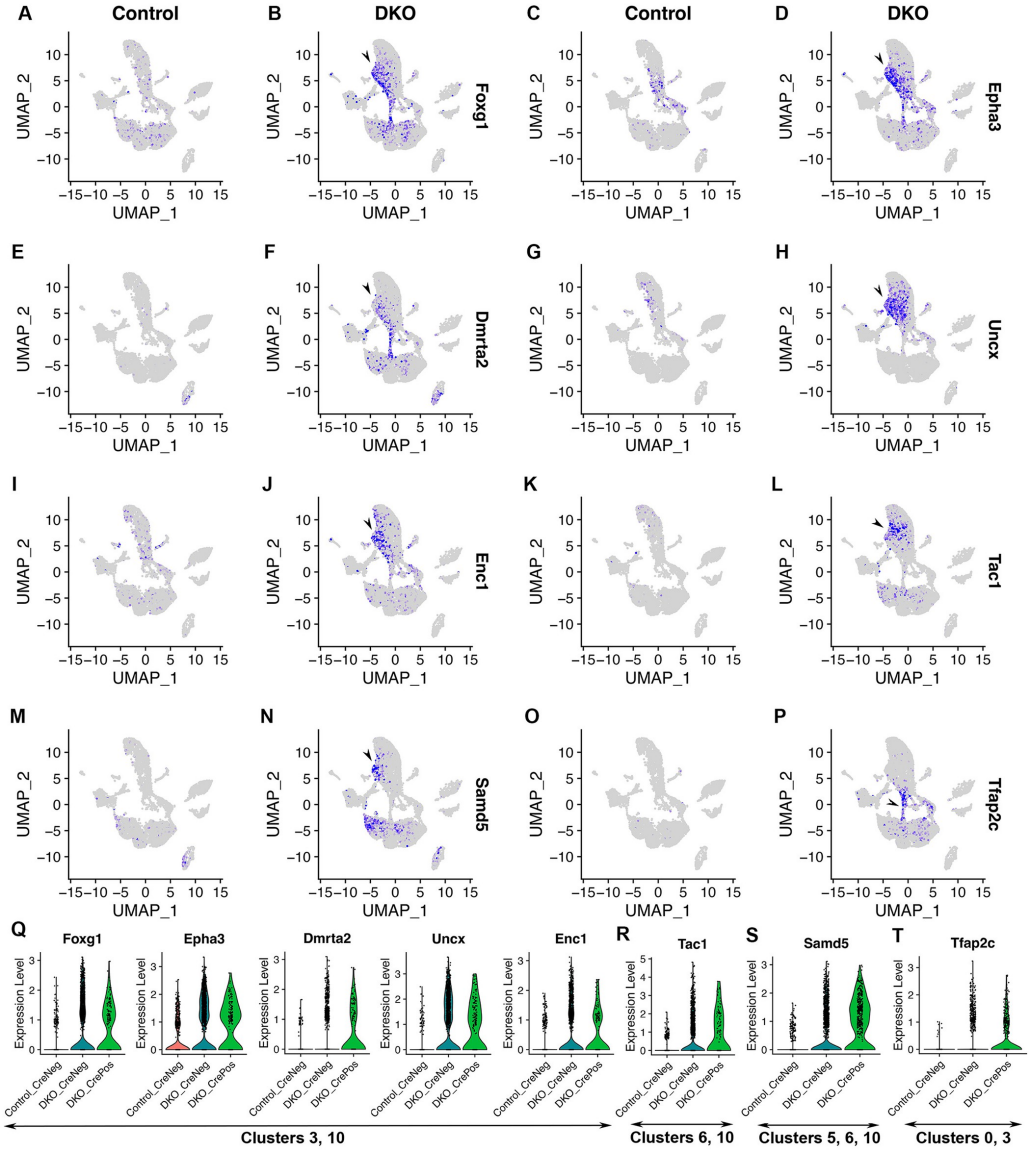
The DKO-specific cell population is featured by gene markers for the forebrain and amacrine cells. See Fig 1 for the information on cell clusters, cell cycle phases, and Six3 and Six6 dual deficiency. **(A–P)** Feature plots showed representative upregulated DEGs that were identified when DKO_CrePos cells and control cells in clusters 10, 6, and 3 were compared (see also S6 Table). In control retinas, these genes were barely or weakly expressed. In the DKO-specific cell population, these marker genes were significantly upregulated (A–N; see also S6 Table). These gene markers are highly expressed in the forebrain, but their expression in the retina is weak or undetectable (see the genepaint database). Besides the DKO-specific cell population, the expression of *Foxg1*, *Dmrta2*, *Epha3*, *Enc1*, *Tac1*, and *Samd5* was also significantly upregulated in naïve retinal progenitor cells upon Six3 and Six6 dual deficiency. Besides forebrain markers, amacrine cell marker *Tfap2c* was significantly upregulated in a subset of the DKO-specific cell population and naïve retinal progenitor cells upon Six3 and Six6 dual deficiency (O, P; see also S6 Table). **(Q–T)** Expression changes in these genes were also shown in violin plots.

To determine the identities of this DKO-specific cell population, we checked positive DEGs identified by comparing DKO_CrePos cells with control cells in clusters 10, 6, and 3 (S6 Table). *Foxg1*, which is strongly expressed in the forebrain and weakly expressed in the retina of E14.5 mouse embryos [33] (also see Genepaint database), was significantly upregulated in the DKO-specific cell population (Fig 4A and 4B; avg_log2FC = 1.1, p_val_adj = 1.66E-54, S6 Table). Similarly, significant upregulation of gene expression was found for *Epha3* (Fig 4C and 4D; avg_log2FC = 0.96, p_val_adj = 7.32E-28, S6 Table), *Dmrta2* (Fig 4E and 4F; avg_log2FC = 0.94, p_val_adj = 8.19E-45, S6 Table), *Uncx* (Fig 4G and 4H; avg_log2FC = 1.7, p_val_adj = 3.41E-89, S6 Table), *Enc1* (Fig 4I and 4J; avg_log2FC = 0.71, p_val_adj = 4.37E-17, S6 Table), *Tac1* (Fig 4K and 4L; avg_log2FC = 1.7, p_val_adj = 5.85E-35, S6 Table), and *Samd5* (Fig 4M and 4N; avg_log2FC = 0.60, p_val_adj = 5.96E-33, S6 Table). In E14.5 mouse embryos, *Dmrta2*, *Epha3*, *Enc1*, *Tac1*, and *Samd5* are highly expressed in the forebrain; *Dmrta2* is modestly expressed in the retina; *Epha3* [34], *Enc1*, and *Tac1* are weakly expressed in the retina; *Samd5* is undetectable in the retina (based on the Genepaint database). In our scRNA-seq dataset, the expression of *Dmrta2*, *Tac1*, and *Samd5* was barely detectable in control retinas (Fig 4E, 4K and 4M). Besides the DKO-specific cell population, the expression of *Foxg1*, *Epha3*, *Dmrta2*, *Enc1*, *Tac1*, *Samd5*, and *Tfap2c* was up-regulated in naïve retinal progenitor cells. Besides these forebrain markers, *Tfap2c*, a gene marker that is expressed in postmitotic amacrine cells in mice [35], was significantly up-regulated in a subset of the DKO-specific cell population (Fig 4Q and 4P; avg_log2FC = 0.67, p_val_adj = 1.94E-37, S6 Table). Expression changes in these genes were also demonstrated in violin plots (Fig 4Q–4T). Taken together, these findings indicate that gene markers for the forebrain and amacrine cells are significantly up-regulated in the DKO-specific cell population.

### Ciliary margin cells are at an edge of the G1-phase naïve retinal progenitor cell cluster in control retinas and this cell population is expanded upon Six3 and Six6 dual deficiency

**Fig 5:**
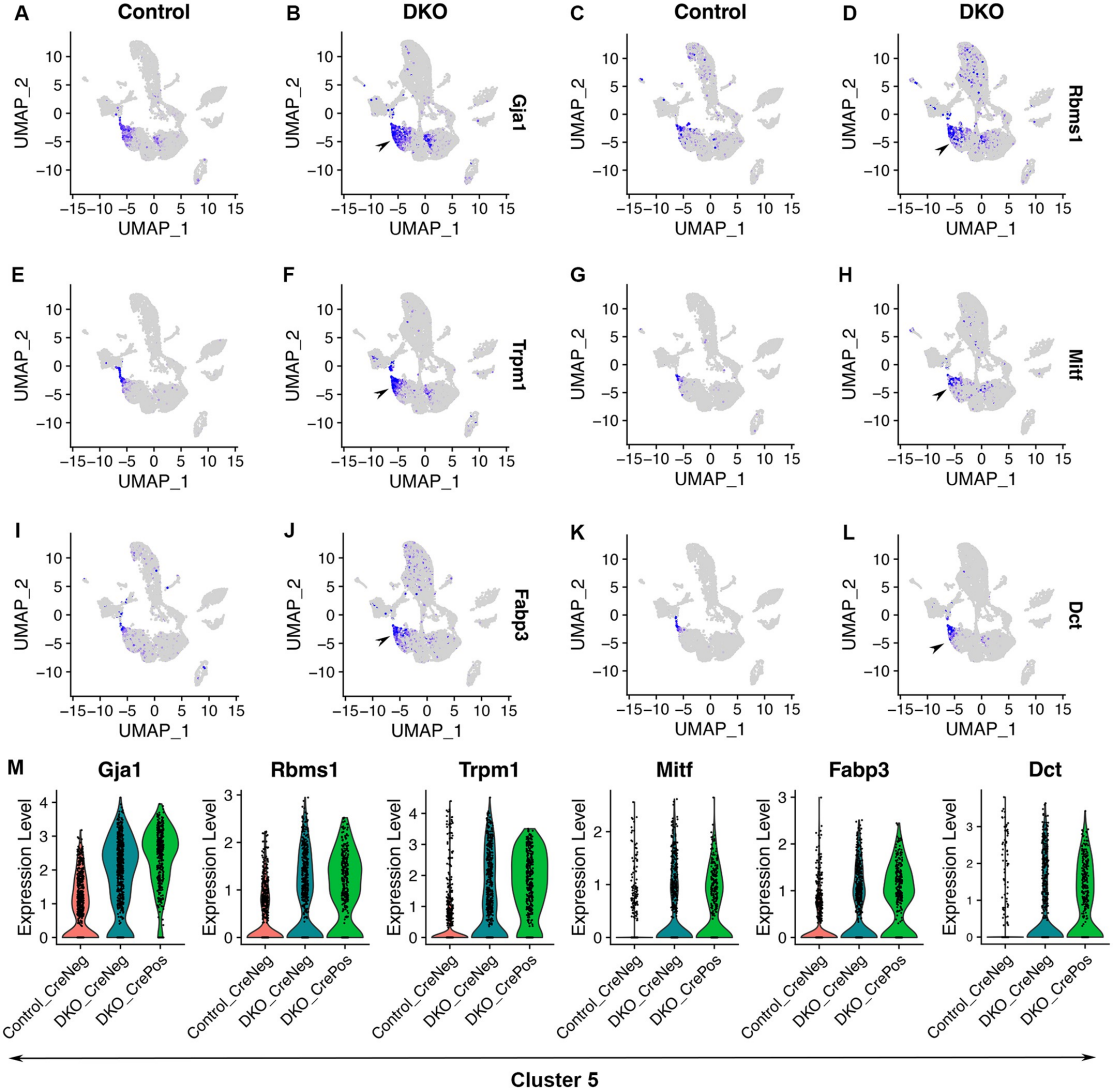
Ciliary margin cells are at an edge of the G1-phase naïve retinal progenitor cell cluster in control retinas and this cell population is expanded upon Six3 and Six6 dual deficiency. See Fig 1 for the information on cell clusters, cell cycle phases, and Six3 and Six6 dual deficiency. **(A–L)** In control retinas, ciliary margin cells were marked by the expression of *Gja1*, *Rbms1*, *Trpm1*, *Mitf*, *Fabp3*, and *Dct*, and these cells were at an edge of cluster 5 (A, C, E, G, I, K, respectively; see also S2 Table). These genes were significantly upregulated, mostly in cluster 5, upon Six3 and Six6 dual deficiency (B, D, F, H, J, L, respectively; see also S7 Table). **(M)** Expression changes of these genes in cluster 5 were also shown in violin plots.

We assessed ciliary margin cells in the scRNA-seq dataset. In control retinas, cells at an edge of cluster 5 (the G1-phase naïve retinal progenitor cell cluster) were marked by the expression of *Gja1*, *Rbms1*, *Trpm1*, *Mitf*, *Fabp3*, and *Dct* (Fig 5; S2 Table). *Gja1* and *Fabp3* are specifically expressed in the ciliary margins of E14.5 mouse embryos (see Genepaint); a homolog of *Trpm1* in zebrafish is expressed in the ciliary margins [36]. *Mitf* is expressed in RPE and ciliary margins [37], and so is *Dct* (see Genepaint). In contrast to these ciliary margin markers, the expression of naïve retinal progenitor cell markers *Ccnd1*, *Sfrp2*, and *Vsx2* was very low at this ciliary margin region in the UMAP graph (Fig 2A, 2C and 2E).

We identified DEGs caused by Six3 and Six6 dual deficiency by comparing DKO_CrePos cells with control cells in cluster 5 (S7 Table). Upon Six3 and Six6 dual deficiency, significant upregulation was found for genes *Gja1* (Fig 5A and 5B; avg_log2FC = 2.1, p_val_adj = 8.07E-90, S7 Table), *Rbms1* (Fig 5C and 5D; avg_log2FC = 1.0, p_val_adj = 6.71E-36, S7 Table), *Trpm1* (Fig 5E and 5F; avg_log2FC = 1.4, p_val_adj = 4.83E-72, S7 Table), *Mitf* (Fig 5G and 5H; avg_log2FC = 0.67, p_val_adj = 2.75E-33, S7 Table), *Fabp3* (Fig 5I and 5J; avg_log2FC = 0.85, p_val_adj = 1.06E-37, S7 Table), and *Dct* (Fig 5K and 5L; avg_log2FC = 1.1, p_val_adj = 1.52E-57, S7 Table). Expression changes in these genes were also demonstrated in violin plots (Fig 5M).

Taken together, the ciliary margin cell population is at an edge of the G1-phase naïve retinal progenitor cell cluster in control retinas and this population expands upon Six3 and Six6 dual deficiency.

### Regulators of the Wnt pathway, FGF pathway, and actin cytoskeleton are affected by Six3 and Six6 dual deficiency

**Fig 6:**
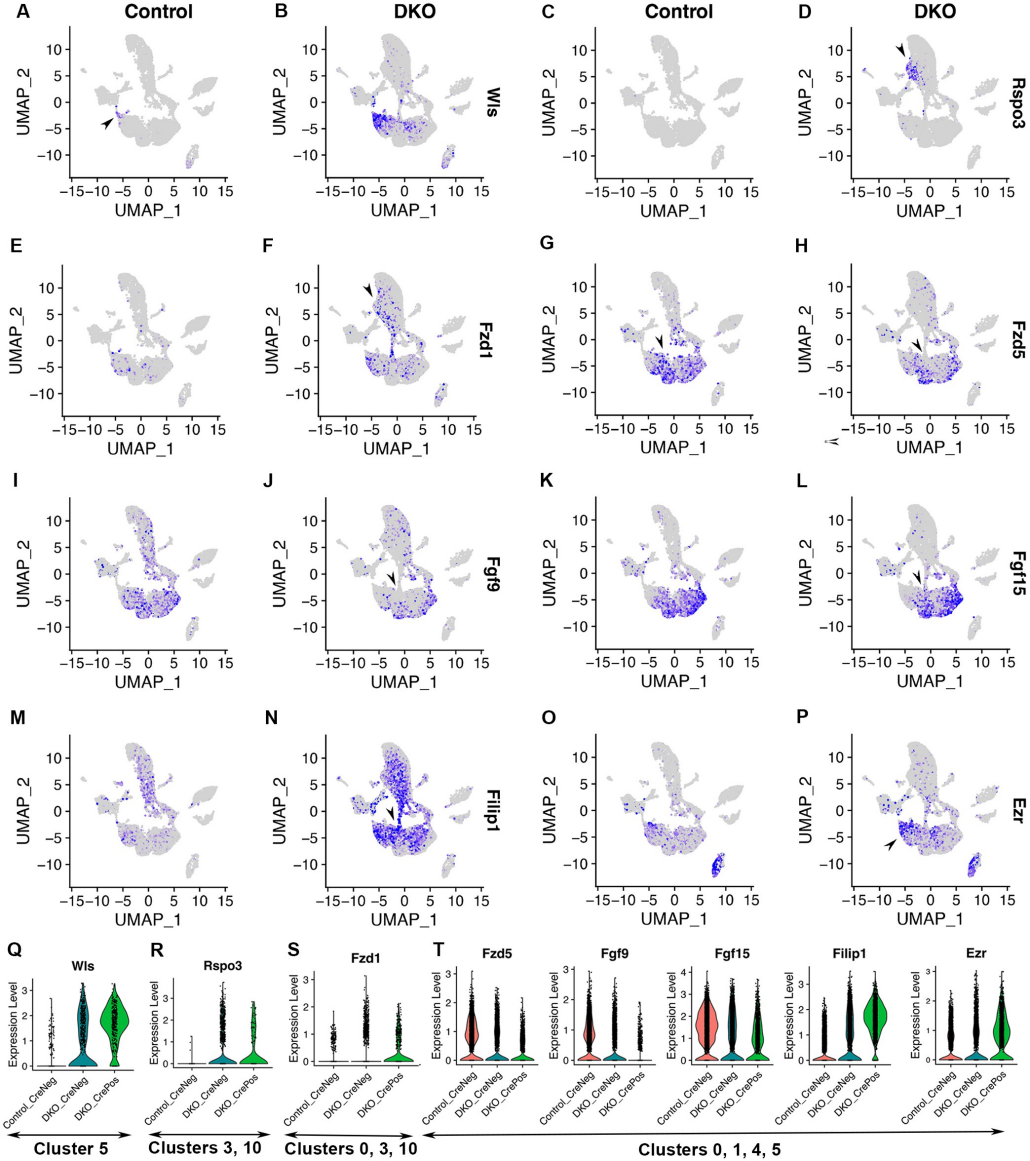
Regulators of the Wnt pathway, FGF pathway, and actin cytoskeleton are affected by Six3 and Six6 dual deficiency. See Fig 1 for the information on cell clusters, cell cycle phases, and Six3 and Six6 dual deficiency. **(A–H)** In control retinas, *Wls* expression was restricted to a subset of ciliary margin cells (A), *Rspo3* expression was undetectable (C), *Fzd1* was sparsely expressed in naïve retinal progenitor cells in cluster 5 (E), and *Fzd5* was widely expressed in naïve retinal progenitor cells (G). Upon Six3 and Six6 dual deficiency, *Wls*, *Rspo3*, *and Fzd1* were significantly upregulated (B, D, F) whereas *Fzd5* was significantly downregulated (H). See also S4–S7 Tables. **(I–L)** In control retinas, *Fgf9* and *Fgf15* were highly expressed in naïve retinal progenitor cells (I, K). In Six3- and Six6-deficient cells, *Fgf9* and *Fgf15* were significantly downregulated (J, L; see also S4 Table). **(M–P)** Actin cytoskeleton regulators *Filip1* and *Ezr* were significantly upregulated upon Six3 and Six6 dual deficiency. See also S4 and S6 Tables. **(Q–T)** Expression changes of these genes were also shown in violin plots.

Our differential expression analysis identified defects in numerous signal transduction pathways following Six3 and Six6 dual deficiency. In the Wnt pathway, *Wls* expression was significantly upregulated in clusters 5 (Fig 6A and 6B, avg_log2FC = 1.6, p_val_adj = 0, S4 Table), and *Rspo3* expression was significantly upregulated in cluster 10 (Fig 6C and 6D, avg_log2FC = 1.1, p_val_adj = 1.47E-56, S6 Table), whereas *Fzd1* expression was widely upregulated in Six3- and Six6-deficient cells (Fig 6E and 6F, avg_log2FC = 0.62, p_val_adj = 5.41E-43, S6 Table). In addition, *Fzd5* expression was significantly down-regulated in naïve retinal progenitor cells (Fig 6G and 6H, avg_log2FC = -0.73, p_val_adj = 1.52E-127, S4 Table). In the FGF pathway, significant downregulation of expression in naïve retinal progenitor cells was found for *Fgf9* (Fig 6I and 6J, avg_log2FC = -0.96, p_val_adj = 7.93E-220, S4 Table) and *Fgf15* (Fig 6K and 6L, avg_log2FC = -0.56, p_val_adj = 9.01E-70, S4 Table), with a stronger reduction in *Fgf9* expression. The expression of *Filip1*, which promotes the degradation of filamin A and therefore remodels the actin cytoskeleton [38], was significantly upregulated (Fig 6M and 6N, avg_log2FC = 1.8, p_val_adj = 0, S4 Table). The expression of *Ezr*, which serves as an intermediate between the plasma membrane and the actin cytoskeleton [39], was significantly upregulated in naïve retinal progenitor cells (Fig 6O and 6P; avg_log2FC = 0.67, p_val_adj = 7.12E-87, S4 Table). These findings indicate that regulators of the Wnt pathway, FGF pathway, and actin cytoskeleton are affected by Six3 and Six6 dual deficiency.

### Validation of major DEGs using in situ hybridization

**Fig 7:**
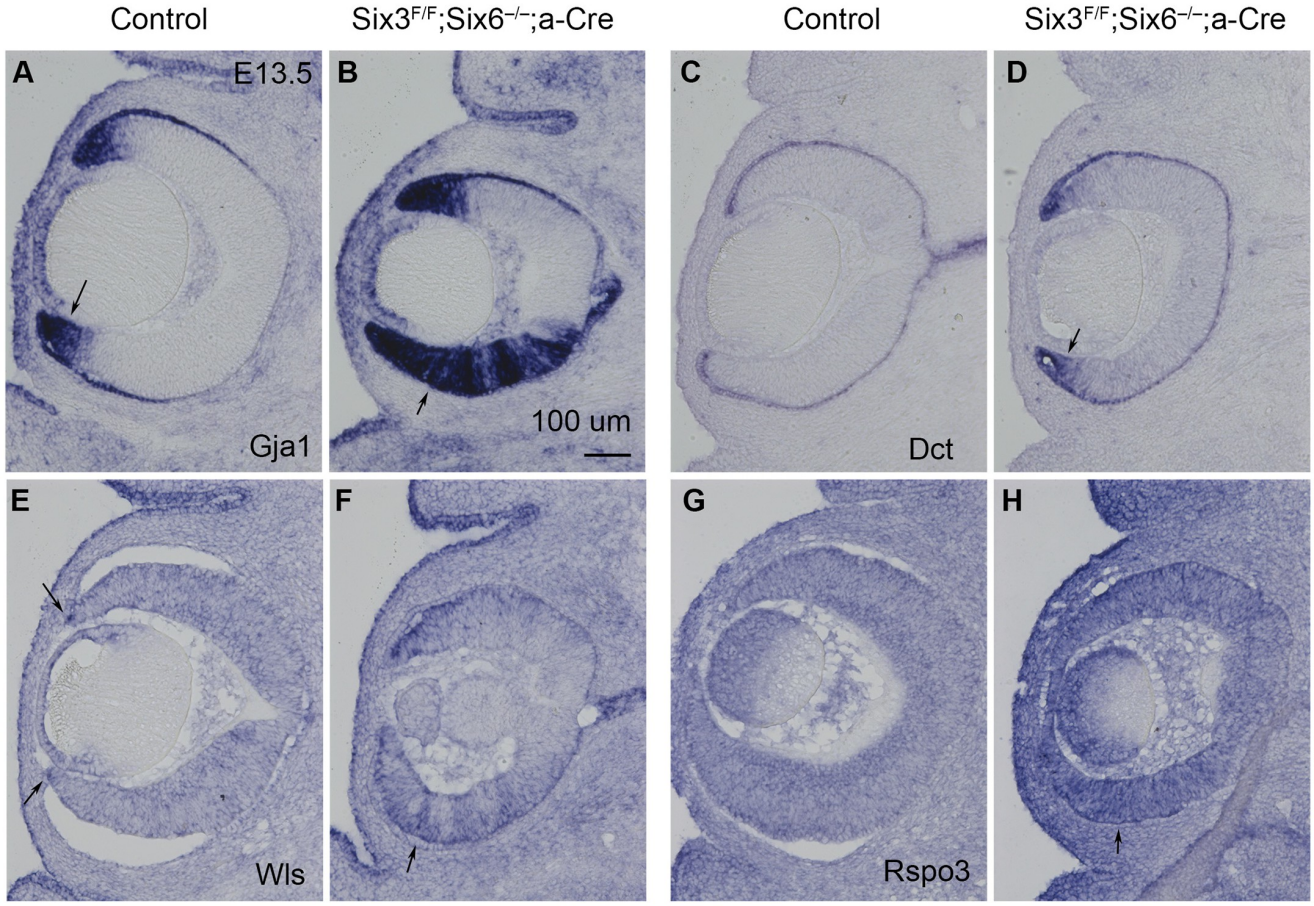
Validation of major DEGs using *in situ* hybridization. **(A, B)** *Gja1* expression was drastically upregulated in peripheral retinas upon Six3 and Six6 dual deficiency. **(C, D)** *Dct* expression was substantially upregulated in peripheral retinas upon Six3 and Six6 dual deficiency. **(E, F)** *Wls* expression was substantially upregulated in peripheral retinas upon Six3 and Six6 dual deficiency. **(G, H)** *Rspo3* expression was substantially upregulated in peripheral retinas upon Six3 and Six6 dual deficiency. Scale bar, 100 μm.

To validate the DEGs identified using scRNA-seq, we performed *in situ* hybridization of DKO and control embryos at E13.5 (Fig 7). In control retinas, *Gja1* was strongly expressed at ciliary margins. In DKO retinas, *Gja1* expression was expanded in peripheral retinas. In control retinas, *Dct* was expressed at the retinal pigment epithelium and the tip of ciliary margins. In DKO retinas, *Dct* expression was drastically upregulated in peripheral retinas. In control retinas, *Wls* was weakly expressed at the tip of ciliary margins. In DKO retinas, *Wls* expression was significantly upregulated in peripheral retinas. In control retinas, *Rspo3* did not show any specific expression above the background level. In DKO retinas, *Rspo3* expression was substantially upregulated in peripheral retinas. Therefore, major DEGs identified using scRNA-seq are confirmed by expression analysis.

### Identification of enriched GO terms in DEGs caused by Six3 and Six6 dual deficiency

**Fig 8:**
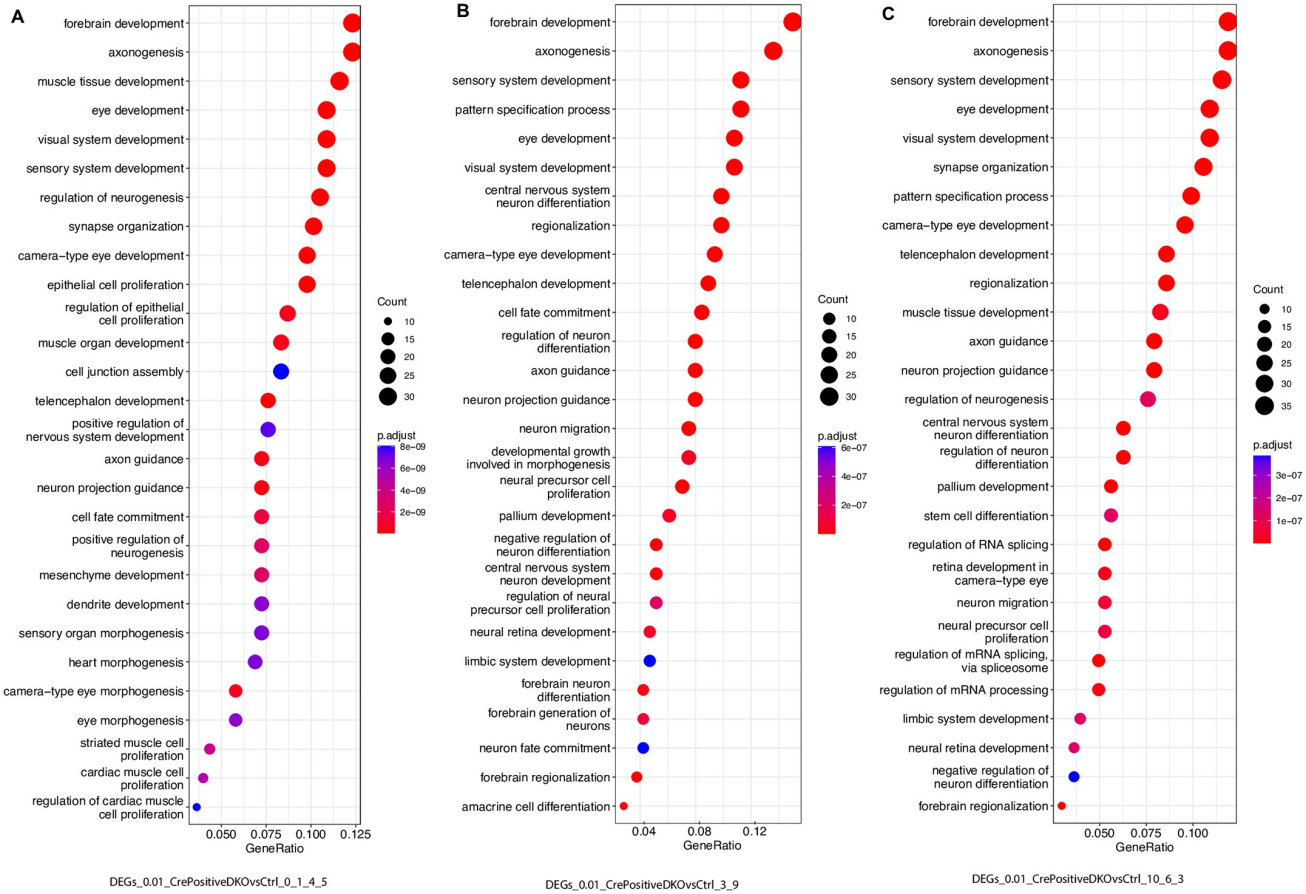
Identification of enriched GO terms in DEGs caused by Six3 and Six6 dual deficiency. **(A)** Enriched GO terms in DEGs caused Six3 and Six6 dual deficiency in naïve retinal progenitor cells (clusters 0, 1, 4, and 5). See also S4 Table. **(B)** Enriched GO terms in DEGs caused Six3 and Six6 dual deficiency in neurogenic retinal progenitor cells (clusters 3 and 9). See also S5 Table. **(C)** Enriched GO terms in DEGs caused Six3 and Six6 dual deficiency in clusters 10, 6, and 3. See also S6 Table.

We functionally annotated the DEGs between DKO_CrePos and control cells in clusters 0, 1, 4, and 5 (S4 Table), in clusters 3 and 9 (S5 Table), and in clusters 10, 6, and 3 (S6 Table). Top enriched GO terms in the DEGs between DKO_CrePos and control cells in clusters 0, 1, 4, and 5 included forebrain development, axonogenesis, eye development, epithelial cell proliferation, and cell junction assembly (Fig 8A). Top enriched GO terms in the DEGs between DKO_CrePos and control cells in clusters 3 and 9 included forebrain development, axonogenesis, eye development, telencephalon development, cell fate commitment, axon guidance, and amacrine cell differentiation (Fig 8B). Top enriched GO terms in DEGs between DKO_CrePos and control cells in clusters 10, 6, and 3 included forebrain development, axonogenesis, eye development, and telencephalon development. Taken together, DEGs in these three groups are enriched with both related and distinct GO terms.

### Developmental trajectories of naïve retinal progenitor cells are altered upon Six3 and Six6 dual deficiency

To elucidate the developmental trajectories of control and Six3- and Six6-deficient retinal cells, we analyzed RNA velocities in the integrated dataset using the scVelo package [28]. The percentages of spliced and unspliced transcripts were 66% and 34%, respectively, for the control sample, and 67% and 33%, respectively, for the DKO sample (S5 Fig), indicating that the capture of nascent transcripts was efficient for both samples. In control retinas, naïve retinal progenitor cells had two major trajectories: one was toward ciliary margin cells in cluster 5, and the other was toward neuronal differentiation through an Atoh7+ neurogenic state. In the neuronal differentiation trajectory, the route toward cluster 7 was a major branch at this stage (S6A Fig). In Six3- and Six6-deficient cells, the trajectory toward ciliary margin cells was enhanced (arrowheads in S6A and S6B Fig), the trajectory toward neuronal differentiation was defective, and an ectopic trajectory lacking the Atoh7+ state led to ectopic neurons (arrows in S6B Fig). Therefore, Six3 and Six6 jointly regulate the developmental trajectories of naïve retinal progenitor cells in the mouse retina.

### Identification of Six3 and Six6 chromatin occupancies at major target genes in wild-type E13.5 mouse retinas

**Fig 9:**
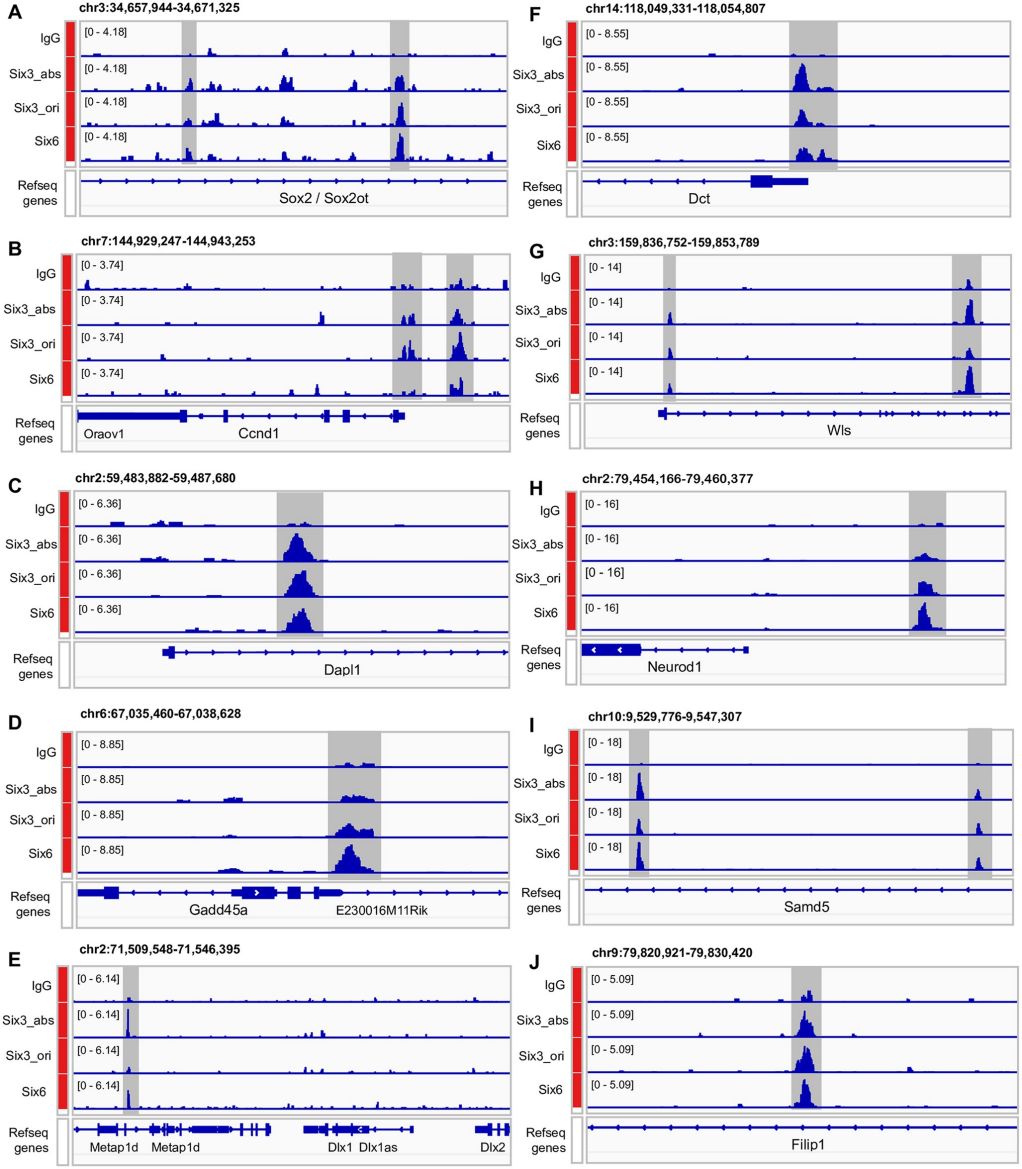
Identification of Six3 and Six6 chromatin occupancies at diverse target genes in multiple cell populations of wild-type E13.5 mouse retinas. Six3 and Six6 chromatin occupancies were identified using CUT&RUN, with two antibodies against Six3 (labeled as Six3_abs and Six3_ori, respectively) and one antibody against Six6. **(A–J)** Six3 and Six6 chromatin occupancies were found at both up-regulated and down-regulated DEGs, and these DEGs were expressed in multiple cell populations of wild-type E13.5 mouse retinas. Notably, multiple peaks of Six3 and Six6 chromatin occupancies were found at *Sox2* (A), which was downregulated upon Six3 and Six6 dual deficiency. Down-regulated DEGs with Six3 and Six6 chromatin occupancies also included naïve retinal progenitor cell marker genes *Ccnd1* (B) and *Dapl1* (C), neurogenic retinal progenitor cell marker gene *Gadd45a* (D), and RGC marker genes *Dlx1* and *Dlx2* (E). Up-regulated DEGs with Six3 and Six6 occupancies included ciliary margin marker gene *Dct* (F), the gene *Wls* that regulates Wnt proteins sorting and secretion (G), neurogenic retinal progenitor cell marker gene *Neurod1* (H), ectopically expressed gene *Samd5* (I), and actin cytoskeleton regulator gene *Filip1* (J).

To assess whether the identified DEGs were direct target genes, we examined Six3 and Six6 chromatin occupancies at these DEGs in wild-type E13.5 mouse retinas. Six3 and Six6 chromatin occupancies were identified using CUT&RUN, with two antibodies against Six3 (labeled as Six3_abs and Six3_ori, respectively) and one antibody against Six6. Chromatin peaks of Six3 and Six6 were called using SEACR1.3 [40] with IgG as a control. Here, we show IGV views for major DEGs; details of the CUT&RUN dataset will be reported elsewhere. Both upregulated and downregulated DEGs had Six3 and Six6 chromatin occupancies. Notably, multiple peaks of Six3 and Six6 chromatin occupancies were found at *Sox2* (Fig 9A), which was downregulated upon Six3 and Six6 dual deficiency [7]. Sox2 is essential for the maintenance of multipotent retinal progenitor cells [14–16]. Down-regulated DEGs with Six3 and Six6 chromatin occupancies also included naïve retinal progenitor cell marker genes *Ccnd1* and *Dapl1* (Fig 9B and 9C), neurogenic retinal progenitor cell marker gene *Gadd45a* (Fig 9D), and retinal ganglion cell marker genes *Dlx1* and *Dlx2* (Fig 9E). Up-regulated DEGs with Six3 and Six6 chromatin occupancies included ciliary margin marker gene *Dct* (Fig 9F), the gene *Wls* that regulates Wnt proteins sorting and secretion (Fig 9G), neurogenic retinal progenitor cell marker gene *Neuorod1* (Fig 9H), ectopic-neuron marker gene *Samd5* (Fig 9I), and actin cytoskeleton regulator gene *Filip1* (Fig 9J). These findings indicate that Six3 and Six6 jointly control diverse target genes that are differentially expressed in multiple cell populations over developmental trajectories of mouse embryonic retinal progenitor cells.

## Discussion

In this study, we performed scRNA-seq of control and DKO mouse eyecups at E13.5. We identified cell clusters based on their transcriptomes and then inferred developmental trajectories based on RNA velocities in the integrated control and DKO datasets. The UMAP graphing of individual cells revealed the relationships between tens of thousands of cells in eyecups. Differential expression analysis not only confirmed previous phenotype studies but also identified cluster markers along developmental trajectories and DEGs caused by Six3 and Six6 dual deficiency. We identified transcriptomes, cell trajectories, and major target genes that are jointly regulated by Six3 and Six6, providing deeper insight into the molecular mechanisms underlying the maintenance and differentiation of retinal progenitor cells.

Cell clustering and RNA velocity analysis reveal developmental trajectories of naïve retinal progenitor cells in control retinas. RNA velocity measures the abundance of unspliced and spliced transcripts, extracting directed information to infer developmental trajectories. In 10x Genomics, cDNA is synthesized using the strategy of annealing oligo dT to stretches of poly A, which is mostly at the 3’-end of genes. In our experiments, cDNA synthesis captured sufficient amounts of unspliced transcripts in our samples for velocity analysis. In the UMAP graph and streamline plot of RNA velocities, naïve retinal progenitor cells progressed toward two major developmental trajectories: one trajectory was toward ciliary margin differentiation, and the other trajectory was toward neuronal differentiation. Cells at the end of the ciliary margin trajectory in the UMAP graph differentially expressed components of the Wnt signaling pathway, such as *Fzd1* and *Wls*. Notably, *Fgf9* and *Fgf15* were expressed in naïve retinal progenitor cells in a pattern that was complementary to that of *Fzd1* and *Wls* in the UMAP graph, forming opposing gradients of Wnt and Fgf activity. Additionally, ciliary margin cells did not express *Atoh7*. The relative positions of cell domains expressing *Fzd1*, *Wls*, and *Fgf15* in the UMAP graph accurately reflected their relative positions in histological sections [8, 41]. Additionally, ciliary margin cells were at an edge of naïve retinal progenitor cell cluster at the G1 phase. These findings strongly indicate that ciliary margin cells are directly differentiated from naïve retinal progenitor cells at the G1 phase under the regulation of the Wnt signaling, consistent with previous findings [5, 7, 8].

Neuronal differentiation from naïve retinal progenitor cells goes through a neurogenic state marked by *Atoh7* expression. *Atoh7* expression overlapped with the expression of naïve retinal progenitor cell markers at one end and early retinal ganglion cell markers at the other end in the UMAP graph, indicating that *Atoh7* expression serves as a transition state toward neuronal differentiation from naïve retinal progenitor cells, consistent with recent findings by the Mu lab [20]. A few key gene markers for retinal ganglion cell differentiation, such as *Pou4f2* and *Isl1*, exhibited a progression of expression patterns toward the end of the retinal ganglion cell trajectory in the UMAP graph. Key gene markers for photoreceptor, amacrine, and horizontal cell differentiation displayed expression patterns that branched out from Atoh7+ neurogenic retinal progenitor cells in the UMAP graph. These findings indicate that neuronal differentiation from naïve retinal progenitor cells goes through a common neurogenic state marked by Atoh7 expression and then toward lineage-specific differentiation through a progression of cell states dictated by a cascade of regulators.

Previous lineage-tracing studies identified a cell lineage from ciliary margin cells to retinal neurons [6, 42, 43], but this lineage was not evident in our cell trajectory analysis of scRNA-seq, possibly due to the sensitivity of the scVelo analysis. Consistently, this lineage was not detected in another cell trajectory analysis of scRNA-seq of the retina [44].

scRNA-seq enables the identification of DEGs caused by Six3 and Six6 dual deficiency in defined cell groups, which is a substantial advantage over bulk RNA-seq. Our previous bulk RNA seq analysis demonstrated that *Six3* and *Six6* compound inactivation, but not any single *Six3* conditional inactivation or *Six6* germline inactivation in naïve retinal progenitor cells, cause major expression changes (Diacou et al., 2018). In our scRNA-seq, we identified DEGs caused by Six3 and Six6 dual deficiency in naïve retinal progenitor cells, neurogenic retinal progenitor cells, the ectopic cell cluster, and ciliary margin cells, respectively; representatives of these DEGs were validated using *in situ* hybridization and supported by chromatin mapping of Six3 and Six6 in E13.5 retinas. A substantial number of DEGs identified by scRNA-seq, such as *Gadd45a*, *Rspo3*, *Enc1*, and *Dlx1* shown in feature plots, were not identified in previous bulk RNA-seq due to the lack of cell clustering [7]. On the other hand, scRNA-seq missed out on some DEGs identified by previous bulk RNA-seq owing to the read depth. A union of DEGs identified by scRNA-seq and bulk RNA-seq revealed over 2000 candidate genes downstream of Six3 and Six6 joint functions. Importantly, a large portion of identified DEGs had Six3 and Six6 chromatin occupancies. Therefore, DEGs identified using scRNA-seq are strongly supported by independent assays.

Major target genes of Six3 and Six6 include *Sox2*, which is a well-known essential regulator of multipotent retinal progenitor cells. Direct transcriptional regulation of *Sox2* at least partially explains the phenotypes in *Six3* and *Six6* compound mutant retinas, but it is unlikely that Six3 and Six6 functions are only passed to Sox2. Indeed, diverse target genes in multiple cell populations across developmental trajectories indicate multiple functions of Six3 and Six6 during early retinal differentiation.

Six3 and Six6 together are essential for the maintenance and proper differentiation of retinal progenitor cells. When Six3 was conditionally deleted in peripheral naïve retinal progenitor cells starting at E10.5 together with Six6 germline inactivation, both naïve and neurogenic retinal progenitor cells exhibited deficits in marker expression: *Ccnd1*, *Sfrp2*, *Vsx2*, and *Atoh7* were significantly downregulated whereas *Neurod1* was significantly upregulated. One important aspect of Six3 and Six6 joint functions was the maintenance of naïve retinal progenitor cells in a proliferative state, as Six3 and Six6 dual deficiency caused downregulation of *Ccnd1*, a lower proportion of cells in the S phase, and a higher proportion of cells in the G1 phase. Additionally, neuronal differentiation was disrupted whereas ciliary margin differentiation was enhanced. Notably, an ectopic cell population that displayed the upregulation of amacrine and forebrain markers was observed. Six3 and Six6 are jointly required for the maintenance and proper differentiation of naïve retinal progenitor cells and the suppression of alternative cell states.

Six3 and Six6 jointly balance the opposing gradients of FGF and Wnt signaling in the maintenance and proper differentiation of naïve retinal progenitor cells. Previous studies indicated that opposing gradients of FGF and Wnt signaling regulate the patterning of eyecups along the central-peripheral axis [8], but how these gradients are regulated is unclear. Downregulation of *Fgf9* and *Fgf15* and the upregulation of *Fzd1*, *Rspo3*, and *Wls* in Six3- and Six6-deficient cells establish that Six3 and Six6 jointly regulate opposing gradients of FGF and Wnt signaling in the central-peripheral patterning of the mouse retina, enabling proper neuronal and ciliary differentiation.

Six3 and Six6 jointly repress the expression of actin cytoskeleton regulators, including *Filip1* and *Ezr*. *Filip1*, which promotes the degradation of filamin A and therefore remodels the actin cytoskeleton [38], was one of the most upregulated genes caused by Six3 and Six6 dual deficiency. The upregulation of *Filip1* and *Ezr* likely remodeled the actin cytoskeleton, which explains the change in cell shapes from elongated retinal progenitor cells to shortened ciliary margin cells upon Six3 and Six6 dual deficiency.

Target regulation by Six3 and Six6 joint functions is dependent on the chromatin context. Some downstream genes, such as *Fzd1* and *Filip1*, displayed wide alterations in expression in Six3- and Six6-deficient cells. Other downstream genes, such as *Neurod1* and *Dct*, exhibited expression changes only in subsets of Six3- and Six6-deficient cells. All these genes had Six3 and Six6 chromatin occupancies, indicating that they are direct target genes. These findings indicate that the regulation of target genes by Six3 and Six6 is dependent on the chromatin context, which could be related to interacting proteins and chromatin states in individual loci. Further studies are needed to elucidate how Six3 and Six6 jointly regulate their target genes context-dependently.

Taken together, our scRNA-seq analysis of control and DKO eyecups identifies cell states and trajectories in control retinas and unravels how these cell states and trajectories change upon Six3 and Six6 dual deficiency. Our scRNA-seq confirms previous phenotypic analyses as well as provides novel insight into the mechanisms underlying early retinal differentiation. Our phenotypic analysis, differential expression analysis, and chromatin mapping assays support a model in which Six3 and Six6 jointly activate or repress diverse target genes in multiple cell populations over developmental trajectories of retinal progenitor cells in mice.

### Limitations of the study

One challenge in this study was that DKO retinas comprised Six3-deficient cells and small numbers of Six3-wildtype-like cells, which resulted from the confounding feature of *α-Cre* that was active in the peripheral regions but inactive in the central region of the retina. The challenge was mitigated when DKO_CrePos cells and control cells were compared to identified DEGs caused by Six3 and Six6 dual deficiency. One limitation of using DKO_CrePos cells for the comparisons was the dropout of Cre expression in some DKO cells, a common issue for scRNA-seq. Nevertheless, DKO_CrePos cells in our dataset were proved to be sufficient for differential gene expression analysis. Furthermore, Six3-deficient and Six3-wildtype-like cells exhibited distinct levels of *Six3* mRNA expression, resulting in the delineation of Six3-deficient areas in the UMAP graph. The delineation of Six3-deficient cells enabled us to associate expression changes with Six3 and Six6 dual deficiency, further mitigating the complexity of *α-Cre* activity. Notably, the areas of DKO_CrePos cells and the areas with strong reductions in *Six3* mRNA expression in the UMAP graph matched well, indicating that the Cre activity was highly concordant with conditional *Six3* deletion. If sorted *α*-Cre/GFP+ cells are used for cell capture, the loss of small cell populations and a significant increase in cell death are inevitable. Complete conditional *Six3* deletion together with germline *Six6* deletion in mouse naïve retinal progenitor cells is desirable, but we are unaware of such a Cre mouse line. Conditional *Six3* deletion using transgenic Six3-Cre together with *Six6* deletion ablates the neuroretina [45], eliminating the possibility of studying the multipotency of retinal progenitor cells. Future development of better mouse Cre lines will bring functional studies of retinal genes to the next level.

## Materials and methods

### Mice

*Six3* and *Six6* double knockout mice (*Six3*^*F/F*^ *Six6*^*−/−*^ *α-Cre*) were described in our previous studies [7]. The Animal Use Protocol 00001259 was approved by the Institutional Animal Care and Use Committee at Albert Einstein College of Medicine. Mice used in this study are in the category of tissue harvest after euthanasia, which is done by CO2 overdose to the effect followed by cervical dislocation. To alleviate suffering, mice that appeared ill due to old age or other non-experimental reasons were euthanized.

### Cell capture from eyecups for scRNA-seq

Mouse embryos from the mating between *Six3*^*F/F*^ *Six6*^*+/−*^ *α-Cre* males and *Six3*^*F/F*^ *Six6*^*+/−*^ females were harvested at E13.5. Intact eyecups containing the neural retina and lens were dissected from neighboring tissues. Eyecups from an embryo with the genotype of *Six3*^*F/F*^ *Six6*^*−/−*^ *α-Cre* were identified based on GFP-positive rosettes in the retinas under a stereo fluorescence microscope. The correlation between GFP-positive rosettes and the genotype of *Six3*^*F/F*^ *Six6*^*−/−*^ *α-Cre* was previously established in pilot studies. Eyecups with normal morphology from an embryo without GFP fluorescence were used as the control. Tails of the selected embryos were collected for genotyping. The eyecups were then dissociated into single cells using activated papain (Worthington Biochemical, NJ, USA) for cell capture using the Chromium fluid device (10x Genomics, CA, USA), targeting 10,000 cells for each sample. Genotyping of the embryos confirmed the genotype *of Six3*^*F/F*^ *Six6*^*−/−*^ *α-Cre* for the DKO sample and the genotype of *Six3*^*+/−*^ *Six6*^*+/−*^ for the littermate control. The heterozygous *Six3* null allele (the excised floxed allele) in the absence of *α-Cre* in the control embryo was caused by leaky *α-Cre* activity in germ cells, resulting in sperms carrying a *Six3* null allele, which was found occasionally in breeding. Our previous study indicated that the retinas of embryos with the genotype *Six3*^*+/−*^ *Six6*^*+/−*^ are similar to those in wild-type embryos at this stage [7]. Captured cells were used for library preparation using the 10x Genomics Single Cell 3’ kit (version 3).

### Analysis of scRNA-seq

Single-cell sequencing libraries of the DKO and control retinas were sequenced on a HiSeq lane (GeneWZ, NJ, USA). Sequencing reads were mapped to the mouse reference genome mm10 (3.0.0) using CellRanger (3.1.0).

Quality control and data analysis were further performed using the Seurat package (3.2.0) [26]. Briefly, cells were filtered (nFeature_RNA > 200 & nFeature_RNA < 6000 & percent.mt < 20), resulting in 9822 and 12146 cells for control and DKO cells, respectively. The two datasets were normalized, and their variable features were determined. Anchors of the two datasets were identified for integration using the function *FindIntegrationAnchors*, and the two datasets were integrated using the function *IntegrateData*. The integrated dataset was scaled using the function *ScaleData*. Dimension reduction, graph-based visualization, and clustering were accomplished using functions *RunPCA*, *RunUMAP (dims = 1*:*20)*, *FindNeighbors*, and *FindClusters (resolution = 0*.*5)*. To identify Cre-positive cells in the DKO sample, we mapped sequencing reads from the DKO sample to the alpha-Pax6-Cre-GFP vector [46] and computed the vector-specific counts in individual cells. These counts were then added to the meta.data of the processed Seurat object. DKO retinal cells with two or more Cre counts were assigned as DKO_CrePos cells. Cluster markers (S2 Table) were identified using the function *FindAllMarkers* in Seurat with default statistical settings when one cluster was compared to all other clusters in the integrated dataset. DEGs caused by Six3 and Six6 dual deficiency (S4–S7 Tables) were identified using the function *FindMarkers* with default statistical settings when DKO_CrePos cells and control cells in selected clusters were compared. The S2, S4, S6, and S7 Tables were further filtered by the criteria p_val_adj < 0.01, and the S5 Table had an additional column p_adj_FDR which was added using the function *DEGs$FDR = p*.*adjust(DEGs$p_val*, *method = ’fdr’)*. *FeaturePlot* used arguments *min*.*cutoff = ’q9’*, *max*.*cutoff = ’q90’* for better contrast in displays. Additionally, cluster averages of gene expression were calculated and highly variable genes were detected using the “vst” method in Seurat. These genes were used for the PCA analysis of cluster averages after scaling as well as pairwise correlation and hierarchical clustering analysis. Functional annotations of DEGs (filtered by p_val_adj < 0.01) were achieved using the function *enrichGO* in the clusterProfiler package [47]. The cell trajectory analysis of retinal cells was performed using the scVelo package [28]. Non-retinal cells and cluster 2 (since cluster 2 had low values for nCount_RNA and nFeature_RNA) were removed before the cell trajectory analysis.

### *In situ* hybridization

Mouse embryos from the mating between *Six3*^*F/F*^ *Six6*^*+/−*^ *α-Cre* males and *Six3*^*F/F*^ *Six6*^*+/−*^ females were harvested at E13.5, fixed in 4% paraformaldehyde at 4°C overnight, and then soaked in 30% sucrose for cryopreservation. DKO and control embryos were sectioned at 10 μm for in situ hybridization using DIG-labeled RNA probes (Sigma, MO, USA). DNA templates for making RNA probes were generated using PCR with specific primers (S8 Table). The *genepain*t database for the query of in situ hybridization images was previously reported [48].

### Statistical analysis

Default statistical settings in the functions *FindAllMarkers* and *FindMarkers* were used to generate S2 and S4–S7 Tables. In the main text, the values of p_val_adj (Bonferroni, the default statistical method for the function *FindMarkers* in Seurat) of DEGs were reported except for the gene *Otx2* where p_adj_FDR was reported. For GO term enrichment analysis, qvalueCutoff was 0.05. The results of *in situ* hybridization represented a minimum of three embryos.

## Supporting information

S1 Fig Identification of non-retinal cell clusters in the integrated scRNA-seq dataset. Related to Fig 1. See also S2 Table. See Fig 1 for the information on cell clusters, cell cycle phases, and Six3 and Six6 dual deficiency. **(A–D)** Clusters 8 and 19 differentially expressed *Hba-x* (A, B); clusters 8, 11, and 19 differentially expressed *Hba-a1* (A-D). These findings indicated that clusters 8, 11, and 19 were red blood cells. **(E–H)** Cluster 17 differentially expressed *C1qb* and *Tyrobp*, indicating that these cells were white blood cells. **(I–L)** Clusters 12 and 14 differentially expressed *Cryab* and *Cryaa*, indicating that they were lens cells. R, red blood cells; W; white blood cells; L, Lens cells.
(TIF)

S2 Fig Clusters 2 and 13 are marked by negative gene markers that are also nearly absent in red blood cells and differentiating lens cells. Related to Fig 1. See also S2 Table. See Fig 1 for the information on cell clusters, cell cycle phases, and Six3 and Six6 dual deficiency. **(A–H)** Clusters 2 and 13 barely expressed *Kcnq1ot1*, *mt-Co1*, *mt-Nd2*, and *mt-Nd4*. These gene markers were also nearly absent in clusters 8, 11, and 14.
(TIF)

S3 Fig Violin plots of nCount_RNA, nFeature_RNA, and percent.mt for cell clusters. Related to Fig 1. **(A)** Clusters 2, 13, and 14 had lower values for nCount_RNA. **(B)** Clusters 2, 8, 11, 13, and 14 had lower values for nFeature_RNA. **(C)** Clusters 2, 8, 11, 13, and 14 had lower values for percent.mt.
(TIF)

S4 Fig Both the PCA analysis of cluster averages of gene expression and hierarchical clustering of correlation matrices of the expression indicate that cluster 10 is closest to cluster 6. **(A)** The PCA analysis of cluster averages. **(B)** The hierarchical clustering of the correlation matrix.
(TIF)

S5 Fig Quantities of spliced and unspliced transcripts in control and DKO retinas. (TIF)

S6 Fig Developmental trajectories of naïve retinal progenitor cells are altered upon Six3 and Six6 dual deficiency. Non-retinal cells and cluster 2 (since cluster 2 had low values for nCount_RNA and nFeature_RNA) were removed before the cell trajectory analysis. **(A, B)** In control retinas, naïve retinal progenitor cells had two major developmental trajectories: one was toward ciliary margin cells, and the other was toward retinal neurons through a neurogenic state marked by *Atoh7* expression (A). Upon Six3 and Six6 dual deficiency, the developmental trajectory toward ciliary margin cells was enhanced (arrowhead in B), whereas the developmental trajectory toward retinal neurons was defective. An ectopic trajectory lacking the Atoh7+ state led to ectopic neurons (arrows in B).
(TIF)

S1 Table Proportions of cell clusters in control and DKO samples. Related to Fig 1. Assigned cell types are shown.
(XLSX)

S2 Table Marker genes for cell clusters in the integrated scRNA-seq dataset. Related to Fig 1. These cluster markers were identified when one cluster was compared with all other clusters in the integrated scRNA-seq dataset using the function *FindAllMarkers* in Seurat.
(CSV)

S3 Table Proportions of cells at three cell cycle phases. Related to Fig 1. Non-retinal cells were removed before the counting.
(XLSX)

S4 Table DEGs caused by Six3 and Six6 dual deficiency in combined clusters 0, 1, 4, and 5. Related to Figs 2, 5, and 6. These DEGs were identified using the function *FindMarkers in Seurat* when DKO_CrePos cells and control cells in combined clusters 0, 1, 4, and 5 were compared.
(CSV)

S5 Table DEGs caused by Six3 and Six6 dual deficiency in combined clusters 3 and 9. Related to Fig 2. These DEGs were identified using the function *FindMarkers in Seurat* when DKO_CrePos cells and control cells in combined clusters 3 and 9 were compared.
(CSV)

S6 Table DEGs caused by Six3 and Six6 dual deficiency in combined clusters 10, 6, and 3. Related to Figs 3, 4, and 6. These DEGs were identified using the function *FindMarkers in Seurat* when DKO_CrePos cells and control cells in combined clusters 10, 6, and 3 were compared.
(CSV)

S7 Table DEGs caused by Six3 and Six6 dual deficiency in cluster 5. Related to Fig 6. These DEGs were identified using the function *FindMarkers in Seurat* when DKO_CrePos cells and control cells in cluster 5 were compared.
(CSV)

S8 Table Sequences of PCR primers for generating DNA templates for making DIG-labeled RNA probes. Related to Fig 7.
(PDF)
