# Supplementary figures and images for: Six3 and Six6 jointly control diverse target genes in multiple cell populations over developmental trajectories of mouse embryonic retinal progenitor cells
Source: PLoS One. 2024 Oct 24;19(10):e0308839. doi: 10.1371/journal.pone.0308839 (PMC11500937; doi:10.1371/journal.pone.0308839)

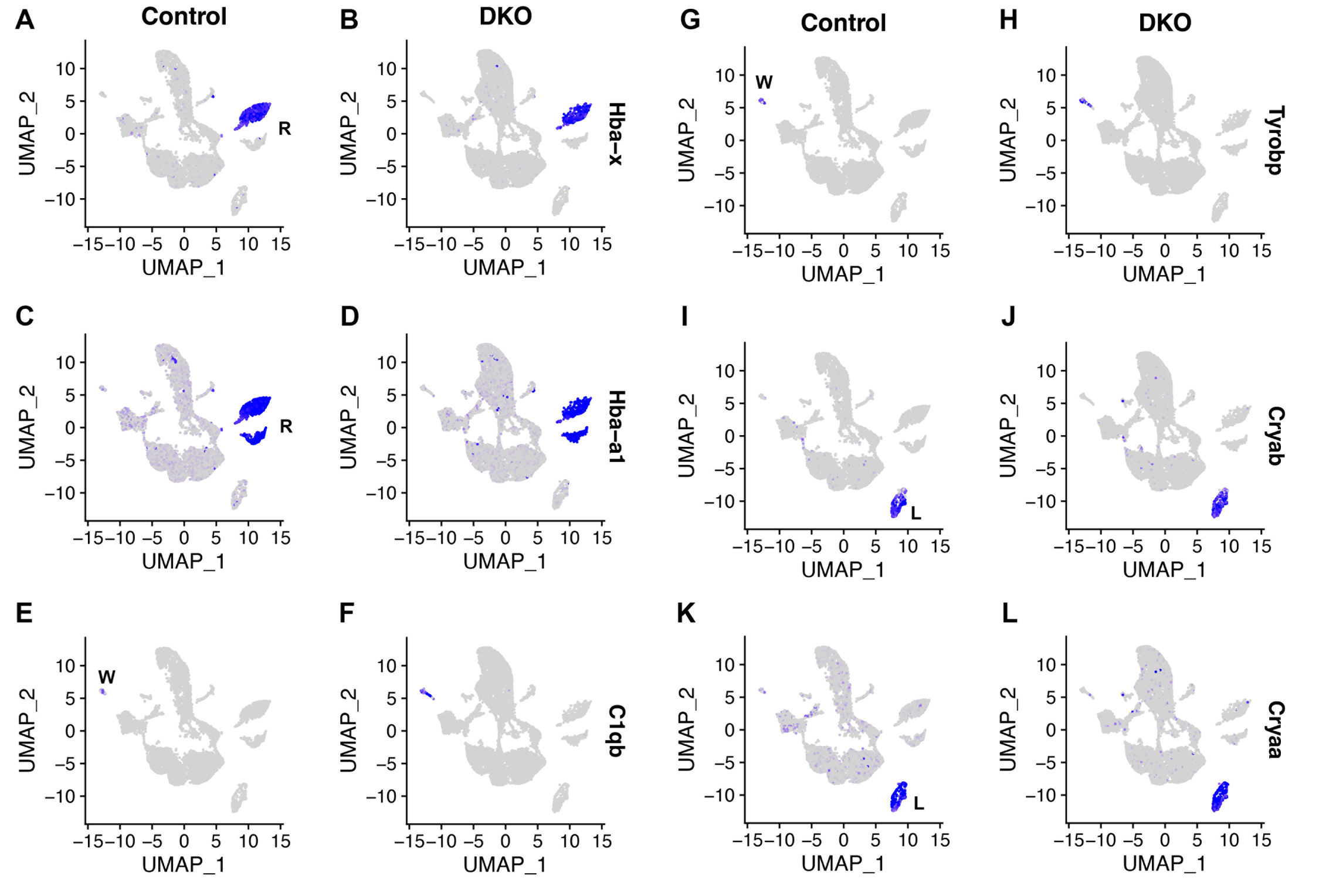

Supplement: S1 Fig — Related to Fig 1. See also S2 Table. See Fig 1 for the information on cell clusters, cell cycle phases, and Six3 and Six6 dual deficiency. (A–D) Clusters 8 and 19 differentially expressed Hba-x (A, B); clusters 8, 11, and 19 differentially expressed Hba-a1 (A-D). These findings indicated that clusters 8, 11, and 19 were red blood cells. (E–H) Cluster 17 differentially expressed C1qb and Tyrobp, indicating that these cells were white blood cells. (I–L) Clusters 12 and 14 differentially expressed Cryab and Cryaa, indicating that they were lens cells. R, red blood cells; W; white blood cells; L, Lens cells. (TIF) [file pone.0308839.s001.tif]

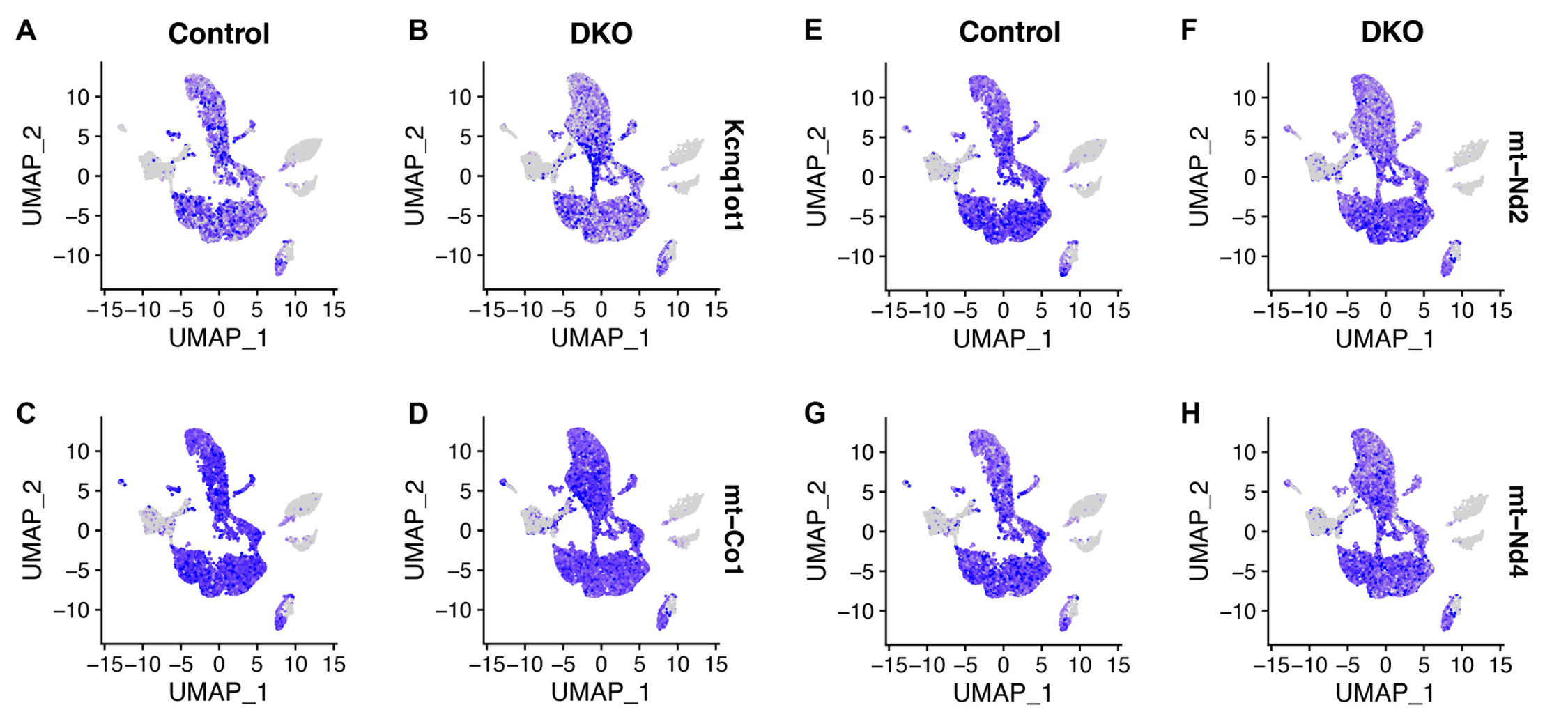

Supplement: S2 Fig — Related to Fig 1. See also S2 Table. See Fig 1 for the information on cell clusters, cell cycle phases, and Six3 and Six6 dual deficiency. (A–H) Clusters 2 and 13 barely expressed Kcnq1ot1, mt-Co1, mt-Nd2, and mt-Nd4. These gene markers were also nearly absent in clusters 8, 11, and 14. (TIF) [file pone.0308839.s002.tif]

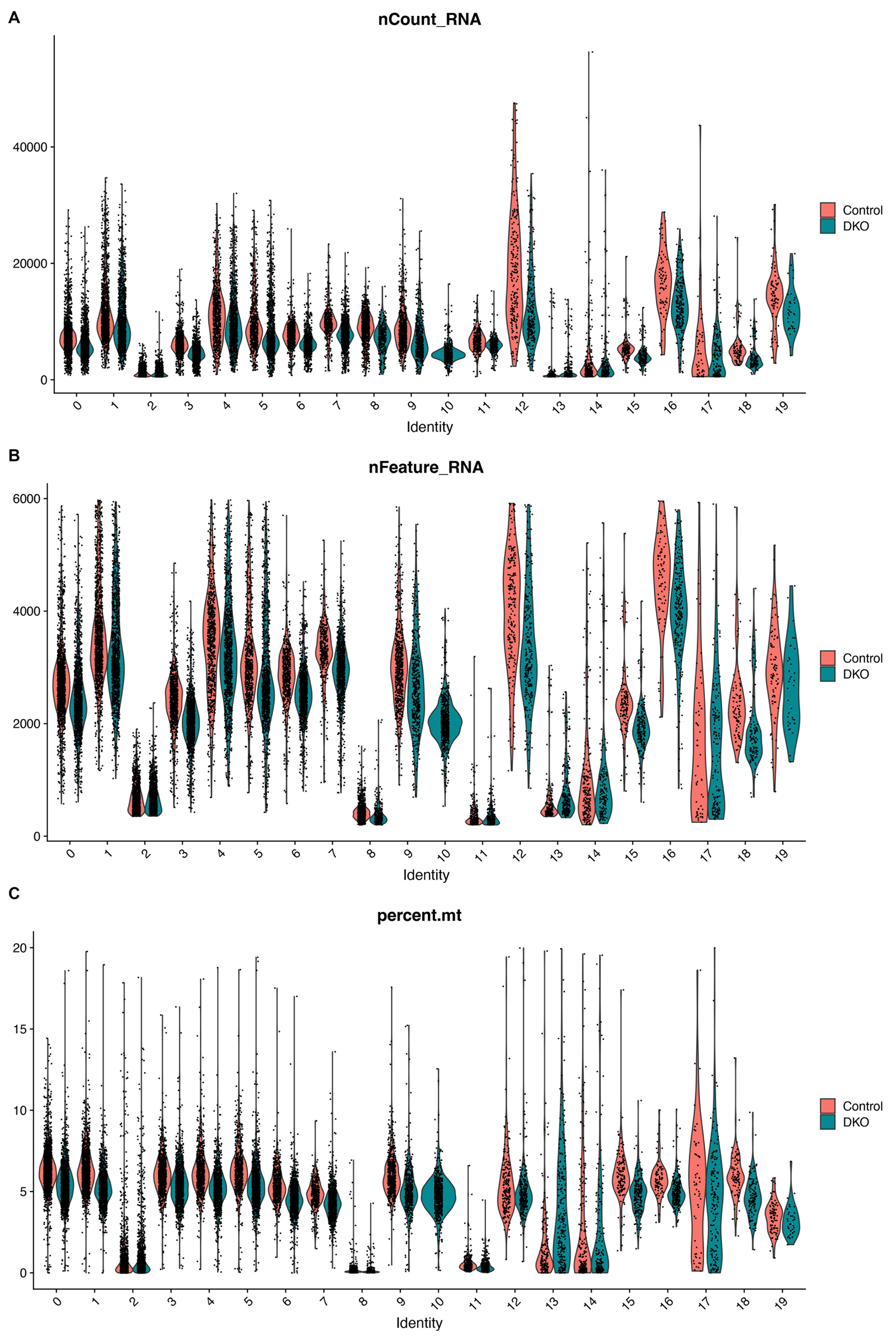

Supplement: S3 Fig — Related to Fig 1. (A) Clusters 2, 13, and 14 had lower values for nCount_RNA. (B) Clusters 2, 8, 11, 13, and 14 had lower values for nFeature_RNA. (C) Clusters 2, 8, 11, 13, and 14 had lower values for percent.mt. (TIF) [file pone.0308839.s003.tif]

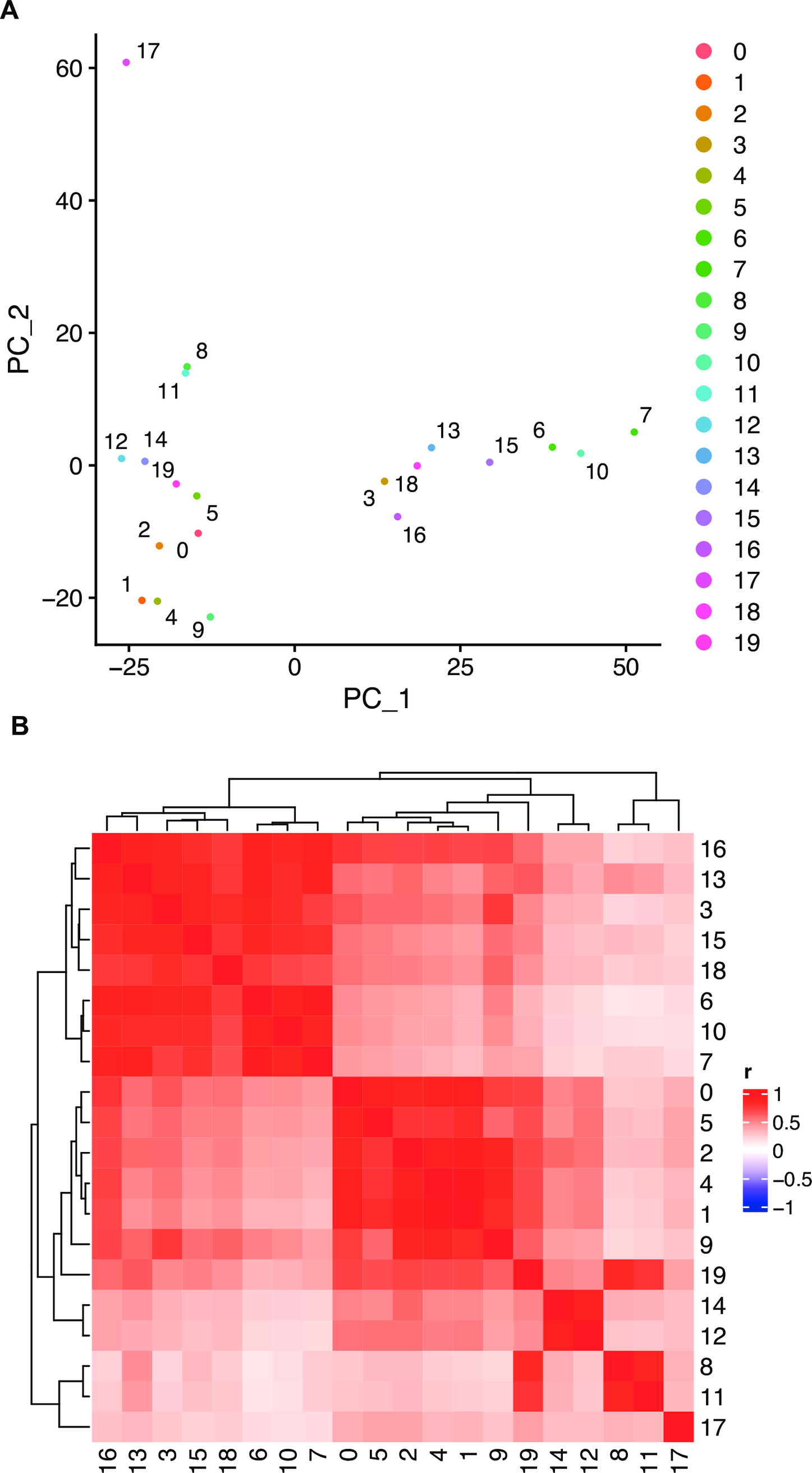

Supplement: S4 Fig — (A) The PCA analysis of cluster averages. (B) The hierarchical clustering of the correlation matrix. (TIF) [file pone.0308839.s004.tif]

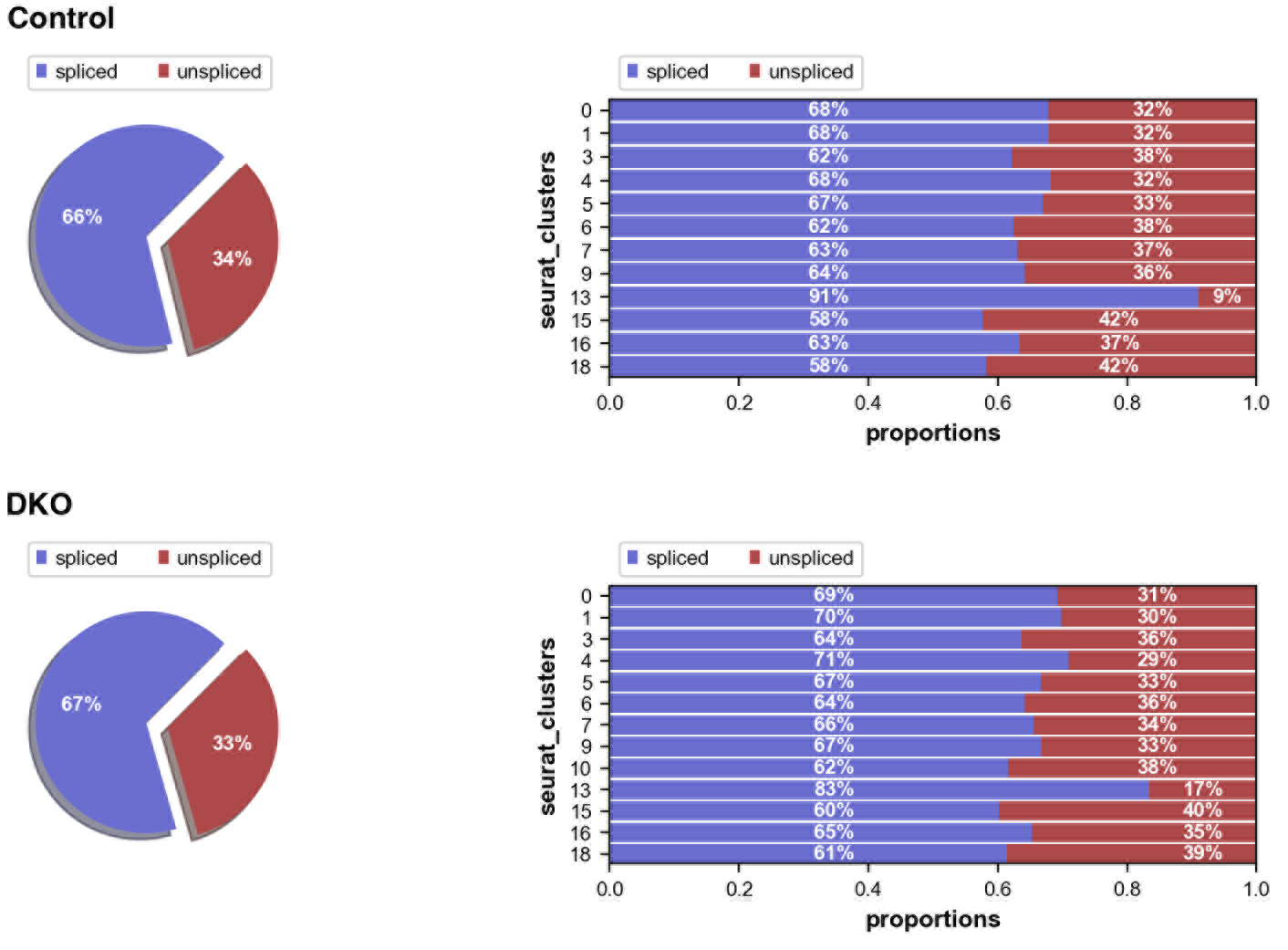

Supplement: S5 Fig — (TIF) [file pone.0308839.s005.tif]

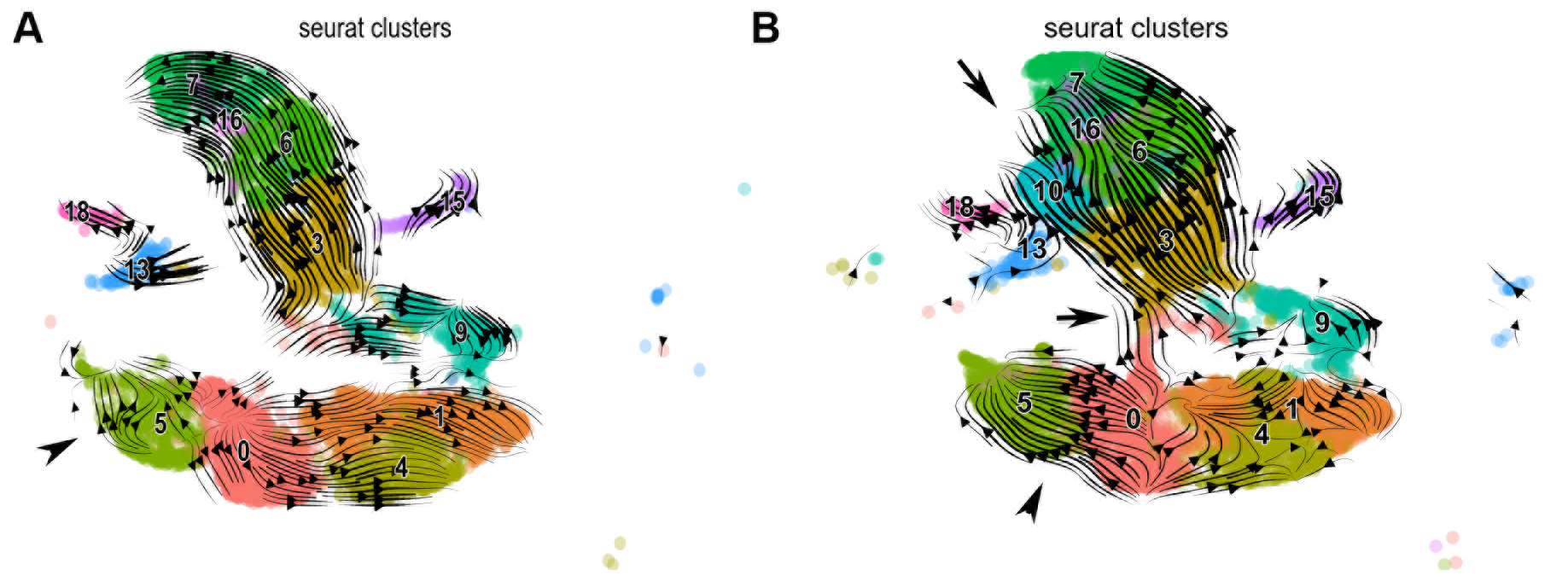

Supplement: S6 Fig — Non-retinal cells and cluster 2 (since cluster 2 had low values for nCount_RNA and nFeature_RNA) were removed before the cell trajectory analysis. (A, B) In control retinas, naïve retinal progenitor cells had two major developmental trajectories: one was toward ciliary margin cells, and the other was toward retinal neurons through a neurogenic state marked by Atoh7 expression (A). Upon Six3 and Six6 dual deficiency, the developmental trajectory toward ciliary margin cells was enhanced (arrowhead in B), whereas the developmental trajectory toward retinal neurons was defective. An ectopic trajectory lacking the Atoh7+ state led to ectopic neurons (arrows in B). (TIF) [file pone.0308839.s006.tif]
