# Supplementary material for: Six3 and Six6 jointly control diverse target genes in multiple cell populations over developmental trajectories of mouse embryonic retinal progenitor cells
Source: PLoS One. 2024 Oct 24;19(10):e0308839. doi: 10.1371/journal.pone.0308839 (PMC11500937; doi:10.1371/journal.pone.0308839)
Supplement: S8 Table — Related to Fig 7. (PDF) [file pone.0308839.s014.pdf]

ID	Sequence	Gene	Size
RD43	cctgcacagccagcattttt	Gja1 UTR #F Mouse In Situ Probe	
RD44	**GAG**taatacgactcactatagggcatttaccagcaccgggact	Gja1 UTR #R Mouse, **RNA Polymerase Binding Site**/T7 Polymerase	814bp
RD47	GTCCGCTTCTTCTGGTGAGT	Dct CDS #F Mouse In Situ Probe	
RD48	**GAG**taatacgactcactatagggaccgtggtgaatgacccaa	Dct UTR #R Mouse, **RNA Polymerase Binding Site**/T7 Polymerase	824bp
RD51	agcctcttcccctggagtag	Wls UTR #F Mouse In Situ Probe	
RD52	**GAG**taatacgactcactatagggcagcacctgggtactccaag	Wls UTR #R Mouse, **RNA Polymerase Binding Site**/T7 Polymerase	845bp
RD177	CCTGCTCAGAACGCCAGAA	Rspo3 mRNA #F Mouse, In Situ Probe	
RD178	**GAG**taatacgactcactatagggTTCTGGGCAACTGTCAAGGC	Rspo3 mRNA #R Mouse, **RNA Polymerase Binding Site**/T7 Polymerase	894bp
